# An ultra-wideband origami microwave absorber

**DOI:** 10.1038/s41598-022-17648-4

**Published:** 2022-08-04

**Authors:** Akash Biswas, Constantinos L. Zekios, Collin Ynchausti, Larry L. Howell, Spencer P. Magleby, Stavros V. Georgakopoulos

**Affiliations:** 1 Department of Electrical and Computer Engineering, Florida International University, Miami, FL 33174 USA; 2 Department of Mechanical Engineering, Brigham Young University, Provo, UT 84602 USA

**Keywords:** Electrical and electronic engineering, Mechanical engineering

## Abstract

Microwave absorbers have been used to mitigate signal interference, and to shield electromagnetic systems. Two different types of absorbers have been presented: (a) low-cost narrowband absorbers that are simple to manufacture, and (b) expensive wideband microwave absorbers that are based on complex designs. In fact, as designers try to increase the bandwidth of absorbers, they typically increase their complexity with the introduction of several electromagnetic components (e.g., introduction of multi-layer designs, introduction of multiple electromagnetic resonators, etc.,), thereby increasing their fabrication cost. Therefore, it has been a challenge to design wideband absorbers with low cost of fabrication. To address this challenge, we propose a novel design approach that combines origami math with electromagnetics to develop a simple to manufacture ultra-wideband absorber with minimal fabrication and assembly cost. Specifically, we utilize a Tachi–Miura origami pattern in a honeycomb configuration to create the first absorber that can maintain an absorptivity above 90% in a 24.6:1 bandwidth. To explain the ultra-wideband behavior of our absorber, we develop analytical models based on the transmission-reflection theory of electromagnetic waves through a series of inhomogeneous media. The ultra-wideband performance of our absorber is validated and characterized using simulations and measurements.

## Introduction

**Figure 1:**
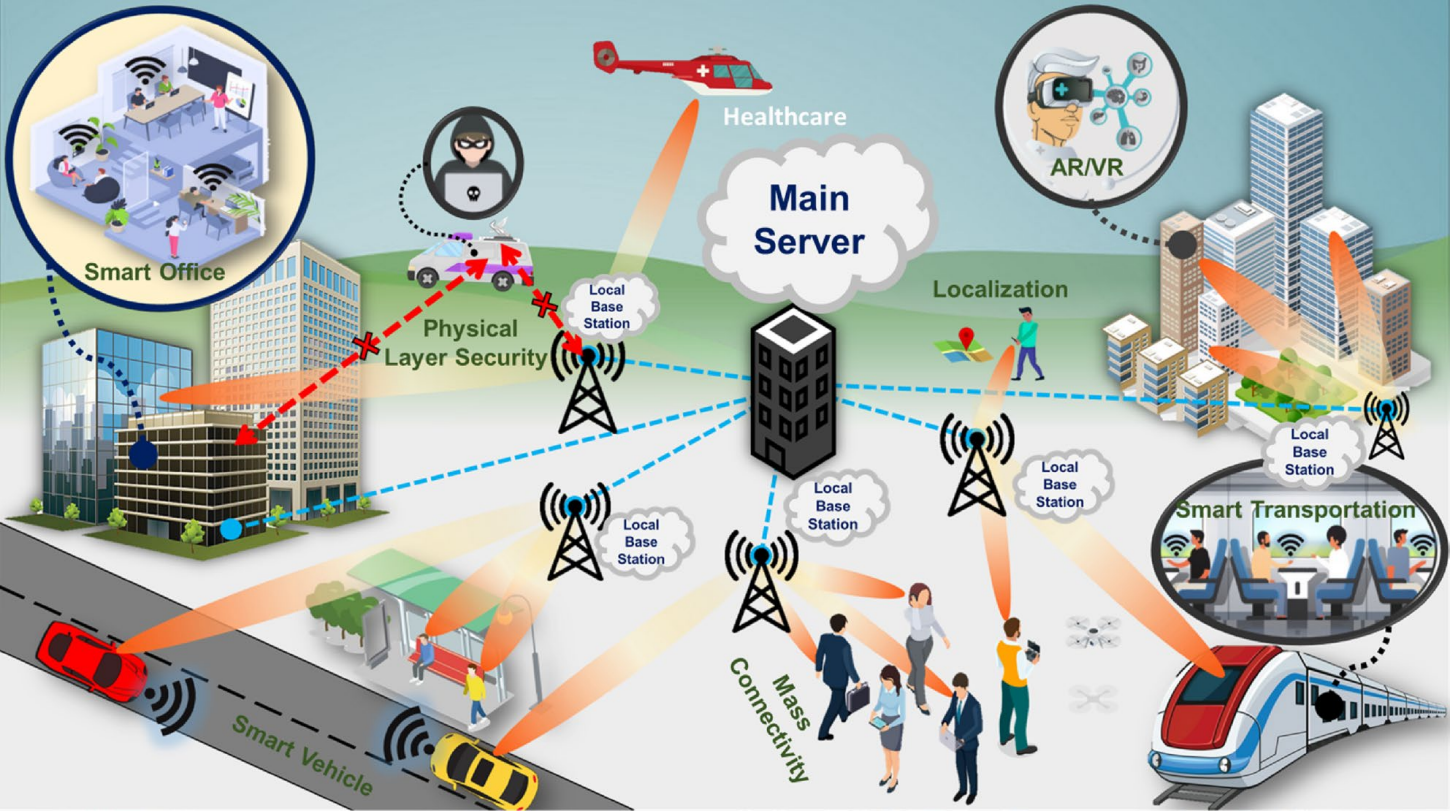
Next-generation communication systems in smart cities.

Next-generation communication systems will enable the development of important future applications that aim to support environments with ubiquitous connectivity. These systems must provide real-time multi-user-to-multi-machine or multi-machine-to-multi-machine communication to connect any business with any service, any computer with any network, and offer immersive connectivity at any place and any time (see Fig. 1). To achieve this, communication systems, which can support high data rates (in the order of 20 Gbps), and high traffic capacities (in the order of 10 Mbps/m^2^), must be developed^1^.

Specifically, it is expected that future applications, such as, virtual reality and augmented reality, will be massively deployed and will need to provide real-time experiences to users with extremely low latencies (e.g., 1 ms)^2^. Certain types of applications of augmented reality (e.g., tele-medicine, tele-surgery, and tele-rescue operations) require highly secure and reliable communications, which can be vulnerable due to the massive number of network users appear and possible interference between different sources. Notably, such interference can be accidental (e.g., destructive interference) or malicious (e.g., network attack). Therefore, electromagnetic shields must be developed to mitigate the effects of interference and protect the integrity of communications.

Significant research has been conducted on devices that can filter and mitigate signal interference. These can be grouped in the following categories: (a) microwave filters at the back-ends of electronic systems, (b) spatial filters, most commonly referred to as frequency selective surfaces (FSSs), at the front-ends of the electronic systems, and (c) engineered structures (e.g., absorbers, metamaterials, etc.) that are used to shield or reduce the radar signatures of electronic systems. This work focuses on the third category by specifically proposing a novel microwave absorber.

Microwave absorbers have been developed since the early 1950s, and significant research has been conducted to study different materials and design approaches^3^. A commonly known narrowband absorber is the Salisbury screen^3^, while other examples include magnetic absorbers^4^, Dallenbach layers^5^, circuit analog absorbers^6^, and Jaumann screens^7^. Also, a wide variety of absorbing materials have been introduced. For example, the pyramidal absorber is widely used in anechoic chambers, which uses a carbon loaded foam and provides broadband operation^8^. Additionally, different material composites have been proposed to provide enhanced microwave absorption^9–12^. Furthermore, with the evolution of 3D printing, additive manufacturing techniques have been recently applied to design absorbers with good electromagnetic performance^13,14^.

Different design methods for absorbers have also been proposed. Kuester et al.,^15^ developed a low-frequency model for the interaction of electromagnetic waves with an array of absorbing rods and wedges. Zadeh et al.,^16^ proposed a simple capacitive circuit method for the fast and efficient design of wideband absorbers. Munk presented simple circuit models for frequency selective surfaces^17^. Other works have explored metamaterials to design absorbers. For example, Landy et al.,^18^ achieved a metamaterial absorber by tuning the effective permittivity and permeability. Sun et al.,^19^ proposed a design of an ultra-wideband absorber based on a destructive interference mechanism. Also, broadband and wide-angle polarization-independent metamaterial absorbers^20^, and absorbers using metasurfaces^21^, have been proposed; such designs manipulate the phase and amplitude of electromagnetic waves^22,23^. Moreover, resistive FSSs have been introduced for the design of broadband absorbers. Pang et al.,^24^ demonstrated a broadband absorber using a high-impedance surface, which comprised of a lossy patch array. Lossy materials, such as resistive sheets^25^, high-loss substrates^26^, lumped resistances^27^, magnetic-materials^28^, and a combination of multiple resonances of different resonators^29^ have also been presented, showing good electromagnetic performance. In addition, various designs of frequency selective surfaces for signal cancellation have been presented. For example, multifunctional active FSSs were proposed by Phon et al.,^30^ and Ranjbar et al.,^31^ these designs achieved a wide range of polarization transformations by cascading subwavelength dielectric gratings.

In summary, significant advancements have been made in the development of microwave absorbers, aiming to produce designs with wide bandwidth and wide-angle insensitivity. However, previous works have either presented highly complex wideband designs with high fabrication costs, or simpler to manufacture narrowband designs. Therefore, it has been a challenge to design absorbers that are both wideband and low-cost. This work aims to address this challenge by developing a novel origami absorber that is ultra-wideband, simple to fabricate, and low-cost.

Origami is the art of paper-folding that has inspired scientists and engineers in different disciplines to develop novel designs. Specifically, origami is able to transform planar sheets to 3D geometries. One of the most impressive and earliest space-borne origami structures was the JAXA Space Solar Flyer (SSF)^32^. Solar arrays^33^, sunshields^34^, and inflatable booms^35^ have also been designed, aiming for space applications. Origami principles have also been used to create designs for biomedical applications, such as microgrippers^36^, medical robots,^37^ and stent grafts^38^. Other origami mechanical deployable structures have also been presented. For instance, Hongbin et al.,^39^ developed mechanical metamaterials with programmable properties by using the self-locking and reconfiguration mechanisms of origami patterns, while Treml et al.,^40^ used an origami waterbomb, demonstrating a 1-bit mechanical storage device that writes, erases, and rewrites itself in response to a time-varying environmental signal. Seymour et al., developed a deployable ballistic barrier using a modified Yoshimura pattern^41^. Mulford et al., used an accordion tesselation to control the radiative heat transfer^42^. In the fields of antennas and microwaves, significant research on origami concepts have also been conducted. Various origami antennas have been introduced by Georgakopoulos et al.,^43–47^ while origami FSSs have been explored by different groups, such as Fuchi et al.,^48,49^ Sessions et al.,^50,51^ Nauroze et al.,^52,53^ and Biswas et al.^54^. An extended review of origami antennas can be found in^55^.

Following our previous work in origami FSSs^54^, in this work, we combine origami math and electromagnetics to design an ultra-wideband absorber. Notably, we are not using origami for its packing capabilities^32^, nor its effect of folding on the electromagnetic performance (e.g., electromagnetic reconfigurability due to folding/unfolding)^45^, which are typically the reasons origami designs are developed. Here, we use origami’s spatial characteristics (in other words, its physical deformation) to enhance the electromagnetic performance of our absorber. Namely, a Tachi–Miura polyhedron (TMP) origami is used to create a novel honeycomb-like design. Honeycomb designs have been extensively used as wideband absorbers^56–60^. Specifically, we show that by using the TMP origami geometry in a honeycomb structure, we create the appropriate physical inhomogeneity, which provides a design with the widest bandwidth ever achieved by a honeycomb absorber. Namely, our absorber maintains an absorptivity above 90% over a 24.6:1 bandwidth; notably, in^61^, we presented preliminary simulation data of our microwave absorber but without explaining its principle of operation as we do here, and without presenting any mechanical analysis, and any measurement data that validate the behavior of our design. It should be pointed out that ultra-wideband origami absorbers have not been reported in the literature before; only the work by Chen et al.,^62^ was recently presented, but only a 5.3:1 bandwidth was achieved.

In what follows, we present the theoretical analysis of our proposed work (“Theory” section), where a thorough study of the electromagnetic (“Electromagnetic analysis” section) and mechanical (“Mechanical analysis” section) behavior of our absorber is performed. In “Methods” section, we present the fabrication process and the measurement setup of our TMP absorber and we compare our measured with our simulated results, showing excellent agreement in the entire bandwidth of operation. Finally, “Discussions” section concludes our work. In the Supplementary Material, the details of our theoretical analysis and 3D design modelling process of our TMP absorber are provided.

## Theory

In what follows, we study the electromagnetic (“Electromagnetic analysis” section) and mechanical (“Mechanical analysis” section) characteristics of our origami TMP absorber, using both analytical and numerical models.

### Electromagnetic analysis

First we prove (“Bandwidth of dielectric slabs backed by ground plane: transmission-reflection theory” section) using an analytical model that a periodic structure of two dielectric slabs backed by a ground plane provides increased absorption compared to a homogeneous structure backed by the same ground plane. Our theoretical results are validated using simulations. In “TMP homogenization method” section, this concept is realized by introducing our origami TMP absorber. To explain the performance of our absorber, an equivalent electromagnetic model is derived. This model is validated in “TMP absorber” section by using simulation analysis.

#### Bandwidth of dielectric slabs backed by ground plane: transmission-reflection theory

Rozanov showed that for a multilayer slab absorber with a prescribed thickness, the largest possible bandwidth is given by:^63^

1 $$\begin{aligned} ln|\Gamma |(\lambda _{max}-\lambda _{min})<2\pi ^2\sum _{i}\mu _{s,i}d_i \end{aligned}$$

where $\Gamma$ is the reflection coefficient in the operating frequency range $f_{min}-f_{max}$ corresponding to wavelength range, $\lambda _{max}-\lambda _{min}$, $\mu _{s,i}$ is the static permeability of the *i*th layer, and $d_i$ is the thickness of the corresponding slab. Based on Eq. (1), it is seen that the bandwidth corresponding to $\lambda _{max}-\lambda _{min}$ is inversely proportional to the reflection coefficient, $\Gamma$. Therefore, the smaller the reflection coefficient is, the wider the bandwidth is. In what follows, we utilize this observation to show that an appropriately defined inhomogeneous domain backed by a ground plane is more wideband than an equally thick homogeneous domain backed by a ground plane.

**Figure 2:**
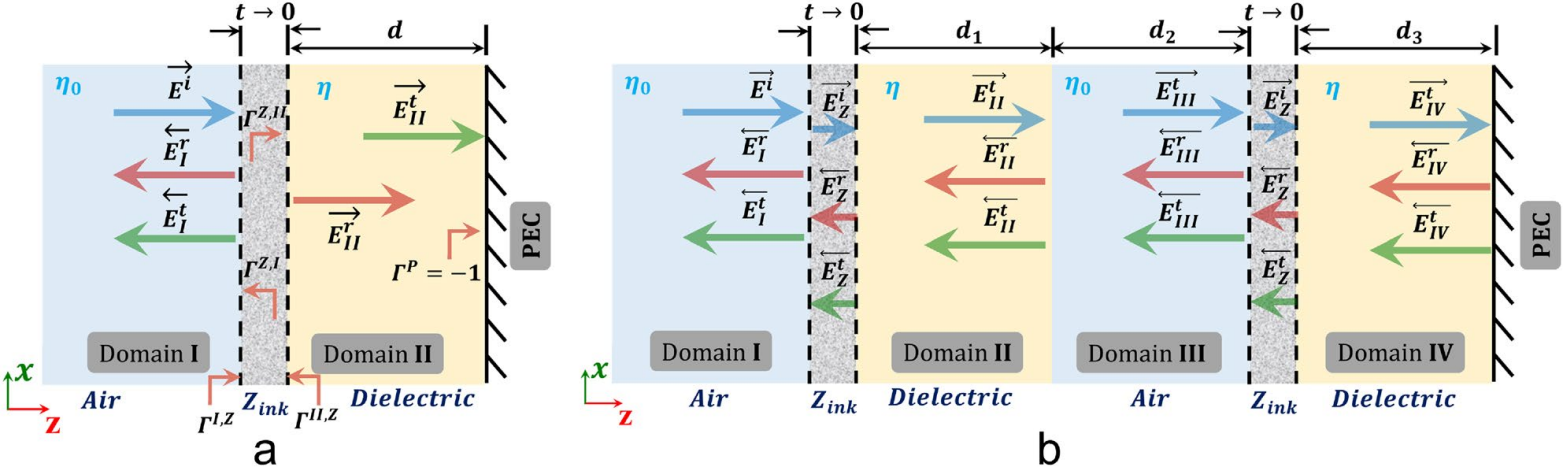
Schematic diagram of media coated with a resistive layer of $Z_{ink}$ impedance. (**a**) Homogeneous medium with one dielectric layer. (**b**) Inhomogeneous medium with two dielectric layers and one air layer.

Figure 2a, and 2b show schematic diagrams of a homogeneous, and an inhomogeneous domain, respectively. The homogeneous domain is comprised of a dielectric layer of thickness, *d*, that is coated with an infinitesimally thin resistive layer with impedance $Z_{ink}$. The inhomogeneous domain is comprised of two layers of dielectric (each one coated with the same infinitesimally thin resistive layer) and one layer of air. The thickness of the inhomogeneous medium is designed to be the same as the thickness of the homogeneous one, thus, $d = d_1 + d_2 + d_3$. The total reflection coefficient for each case can be formulated using electromagnetic theory of transmission-reflection of waves through media. Such formulation is presented in detail in the Supplementary Material. Here, for brevity we show the reflection coefficients for our homogeneous and inhomogeneous media cases:

2 $$\begin{aligned}&\Gamma ^{total}_{Homog.} = \Gamma ^{I,Z} + T^{Z,I} \cdot T^{I,Z}\cdot e^{-j\beta _Zt}(\Gamma ^{Z,II}\cdot e^{-j\beta _Zt} - T^{II,Z}\cdot T^{Z,II}\cdot e^{-2j\beta d}) \end{aligned}$$

3 $$\begin{aligned} \Gamma ^{total}_{Inhomog.} =&{} \Gamma ^{I,Z} + T^{Z,I}\cdot T^{I,Z}\cdot e^{-j\beta _Zt}(\Gamma ^{Z,II}\cdot e^{-j\beta _Zt} + T^{III,II}\cdot e^{-j\beta d}\cdot T^{Z,II}\cdot e^{-2j\beta d}(\Gamma ^{II, III} +\\ &{}T^{III,II}(\Gamma ^{III,Z}\cdot T^{II,III}\cdot e^{-2j\beta _0 d} + T^{Z,III}\cdot e^{-j\beta _0 d}(\Gamma ^{Z,IV}\cdot T^{III,Z}\cdot T^{II,III}\cdot e^{-2j\beta _Zt} \cdot e^{-j\beta _0 d} + \\ &{}\Gamma ^{IV,Z}\cdot T^{Z,IV}\cdot T^{III,Z}\cdot T^{II,III}\cdot e^{-j\beta _Zt}\cdot e^{-j\beta _0 d})))) \end{aligned}$$

**Figure 3:**
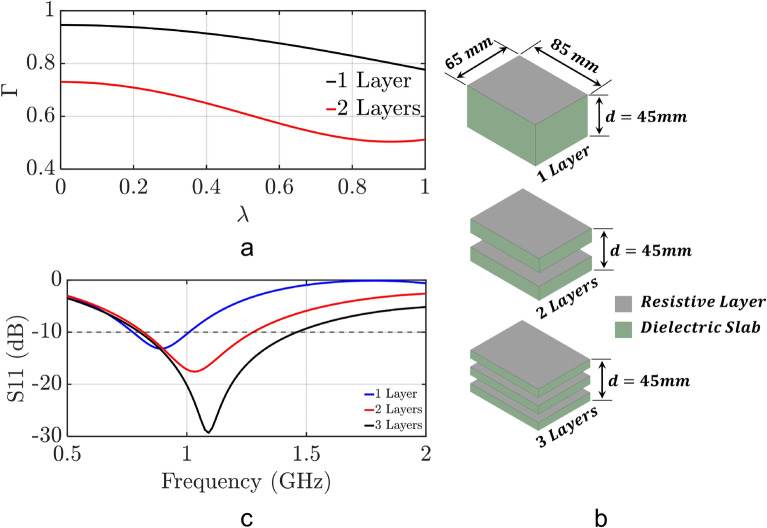
(**a**) Analytically calculated reflection coefficient for the 1-layer homogeneous and 2-layer inhomogeneous media, (**b**) dielectric media studied using simulation, and (**c**) simulated reflection coefficient for the media cases in Fig. 3b.

All the reflection and transmission coefficients in Eqs. (2) and (3) are defined in the Supplementary Material. Next, we evaluate Eqs. (2) and (3) for a range $0-\lambda$, and our results are plotted in Fig. 3a. It is seen that the reflection coefficient of the inhomogeneous medium is lower than the reflection coefficient of the homogeneous medium in the entire range of frequencies. Therefore, based on the relationship (which was explained above) between the reflection coefficient and the bandwidth in Eq. (1), we expect that an absorber that has the same properties with the inhomogeneous medium will exhibit wider bandwidth than an absorber that has the same properties with the homogeneous medium.

To validate our theoretical results, we perform full-wave simulations using ANSYS HFSS of different multi-layer designs: a homogeneous design and two inhomogeneous designs. The homogeneous design consists of an 85 mm long, 65 mm wide, and 45 mm high Polyimide dielectric slab ($\varepsilon _r = 3.5$), as shown in Fig. 3b. A 600 $\Omega {/}\square$ resistive layer is applied on the top face of the slab using an impedance boundary condition, and a perfect electric conducting (PEC) layer is applied on its bottom face using a PEC boundary condition. The first inhomogeneous design consists of an air slab sandwiched between two Polyimide slabs with the same resistive layer on their top faces as the homogeneous design, as shown in Fig. 3b. Also, the lengths and widths of these slabs are identical to the ones of the slab in the homogeneous medium. The thickness of each one of the three slabs (two dielectric slabs and one air slab) is equal to one third of the thickness of the homogeneous slab; therefore, the total thickness of the inhomogeneous design is equal to the thickness of the homogeneous slab, i.e., 45 mm. The second inhomogeneous design consists of two air slabs sandwiched between three Polyimide slabs with the same resistive layer on their top faces as the homogeneous design, as shown in Fig. 3b. The total thickness of the second inhomogeneous design is again equal to 45 mm. The simulated reflection coefficient for all three designs of Fig. 3b are shown in Fig. 3c. Based on these results, it can be concluded that the reflection coefficient of our designs decreases as the number of layers increases, thereby leading to wider absorption behavior. This agrees with the results of our theoretical analysis, which were presented above.

#### TMP homogenization method

**Figure 4:**
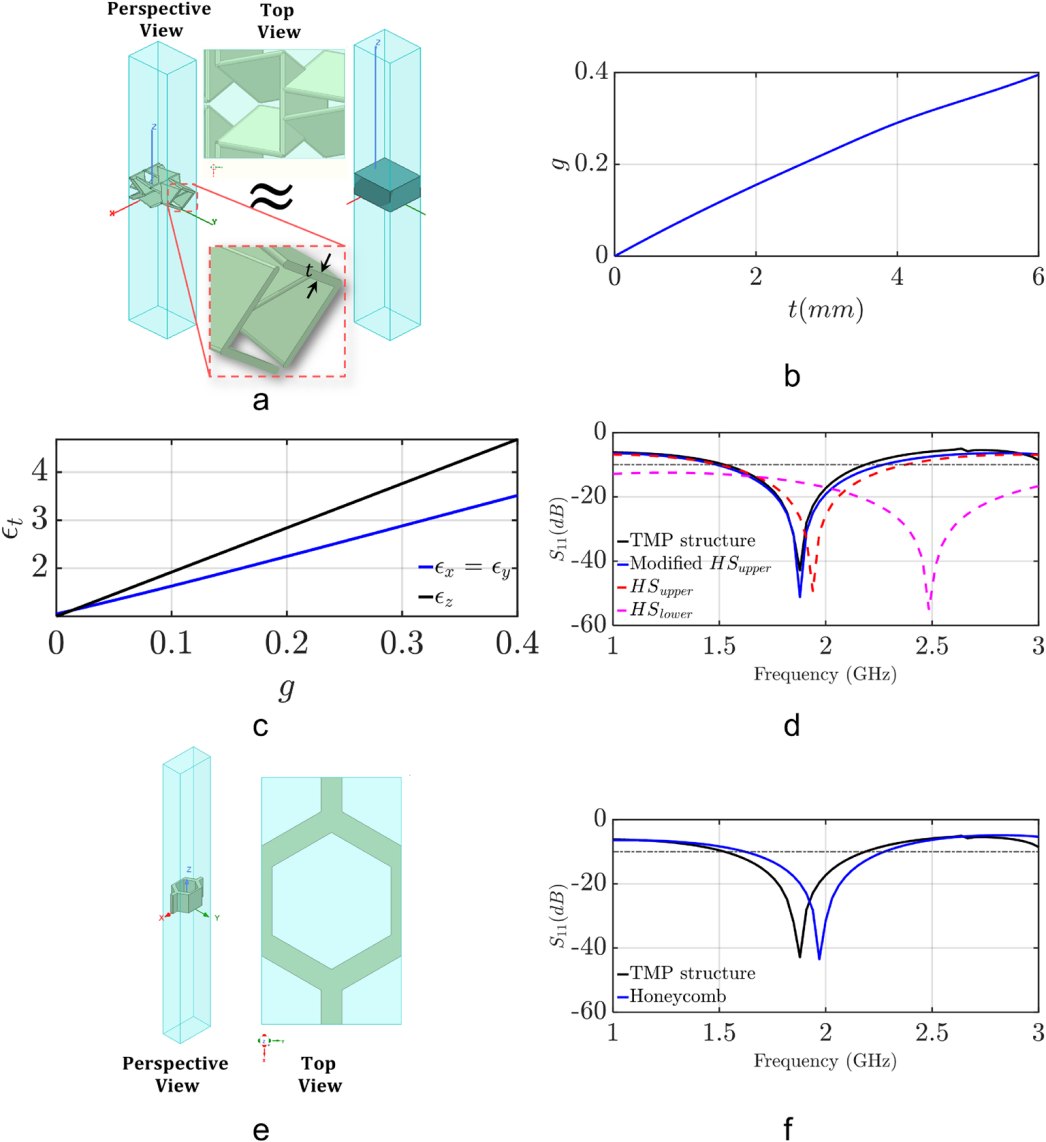
Effective material homogenization analysis of the dielectric structure of our TMP absorber (no metal and resistive layers are included). (**a**) Perspective and top views of our TMP absorber, and its equivalent homogenized model for wall thickness *t* = 4 mm. A step-by step design process for the 3D structure of our TMP absorber is presented in Figs. S4 and S5 of our Supplementary Material in Section 2.2. (**b**) Volume fill factor, *g*, response as the wall thickness, *t*, varies from 0 to 6 mm. (**c**) Relationship between $\varepsilon _t$ and volume fill factor, *g*. (**d**) Evaluated reflection coefficients of TMP absorber following different approaches. (**e**) Perspective and top views of a honeycomb unit cell. (**f**) Reflection coefficients of the honeycomb and TMP unit cell.

In the previous section, we showed that as the number of layers in inhomogeneous designs increases, the bandwidth of effective absorption increases. Next, we aim to find a realistic design that emulates the inhomogeneous structure of Fig. 2b. Various absorbers have been introduced before^57–60,64^. Notably, honeycomb absorbers are typically preferred due to their easy fabrication and wideband performance^57^. Here, we introduce a new honeycomb design that emulates the properties of the multi-layer designs, which were described in “Bandwidth of dielectric slabs backed by ground plane: transmission-reflection theory” section and Supplementary Material Section 1.1. For this purpose, the Tachi–Miura polyhedron (TMP) origami design is used. A detailed description of this origami is provided in “TMP absorber” section. By comparing Figs. 4a (TMP unit cell) and 4e (traditional unit cell of a honeycomb), it can be concluded that a TMP design forms a honeycomb-like structure. Also, the TMP unit cell has discontinuities in the longitudinal direction, thereby providing a multi-layer design (e.g., TMP material and air) like the ones discussed in “Bandwidth of dielectric slabs backed by ground plane: transmission-reflection theory” section.

The performance of honeycomb absorbers is typically characterized by first formulating the effective material properties (i.e., effective permittivity and effective permeability) of their dielectric structure (excluding any metal and resistive layers) as a function of their geometrical characteristics. These formulations lead to semi-analytical models that can quickly and accurately predict the performance of a honeycomb absorber by replacing it with an equivalent medium that has the effective material properties of the absorber^15,65–68^. Therefore, these models are used instead of computationally expensive full-wave simulations to efficiently analyze the performance of honeycomb absorbers. Here, we extend the work by Johansson et al.,^68^ to formulate a semi-analytical model for our TMP absorber by deriving closed-form solutions for its effective electromagnetic properties. Specifically, we apply the homogenization method, to represent our absorber as a uniaxially anisotropic but homogeneous material with tensor permittivity $\varepsilon _t$ and permeability $\mu _t$:

4 $$\begin{aligned} \varepsilon _t = \begin{bmatrix} \varepsilon _x &{} 0 &{} 0\\ 0 &{} \varepsilon _y &{} 0\\ 0 &{} 0 &{} \varepsilon _z \end{bmatrix}, \mu _t = \begin{bmatrix} \mu _x &{} 0 &{} 0\\ 0 &{} \mu _y &{} 0\\ 0 &{} 0 &{} \mu _z \end{bmatrix} \end{aligned}$$

We use the Hashin-Shtrikman (HS) upper and lower bounds^69^ to find the $\varepsilon _i$ and $\mu _i$ components, where *i* = *x*, *y*, which can be used to provide an upper and lower bound for the performance of the absorber:

5 $$\begin{aligned}&\varepsilon _{HS}^L = \varepsilon _0 \frac{(1+g)\varepsilon _a + (1-g)\varepsilon _0}{(1-g)\varepsilon _a + (1+g)\varepsilon _0} \end{aligned}$$

6 $$\begin{aligned}&\varepsilon _{HS}^U = \varepsilon _a \frac{(2-g)\varepsilon _0 +g\varepsilon _a}{g\varepsilon _0 + (2-g)\varepsilon _a} \end{aligned}$$

Equations (5) and (6) require only the material parameters $\varepsilon _0=8.85\cdot 10^{-12}$ $F\cdot {m^{-1}}$, the bulk material value $\varepsilon _a$, and the fill factor *g* that determines the volume fraction of space occupied by material $\varepsilon _a$.

For the case where the periodicity in both *x* and *y* directions is small, compared to the wavelength or the skin depth, the components $\varepsilon _z$ and $\mu _z$ are known exactly^65^:

7 $$\begin{aligned}&\varepsilon _z=(1-g)\varepsilon _0+g\varepsilon _a \end{aligned}$$

8 $$\begin{aligned}&\mu _z=(1-g)\mu _0+g\mu _a \end{aligned}$$

In this analysis, we assume non-magnetic media; therefore, in the rest of our paper, we use $\mu _r=1$. Notably, this choice does not limit our analysis. First, we evaluate the fill factor *g*. Due to the geometrical properties of our design, our fill factor is evaluated for different wall thicknesses *t* using cubic spline interpolation, and is expressed as:

9 $$\begin{aligned} g = -5.585\cdot 10^{-5}t^3-0.00255t^2+0.083t+0.0005426 \end{aligned}$$

Figure 4b shows how the fill factor changes as we vary the wall thickness, *t*, in the range of 0.1–6 mm. By using this fill factor, and for a given $\varepsilon _a$, we apply Eqs. (5) and (6) to find the upper and lower bound responses. Figure 4d shows these analytical results for the TMP absorber and compares them to our simulation results based on ANSYS HFSS. It is seen that the analytically calculated upper bound response does not agree well with the simulated response (which accurately represents the performance of the design). Therefore, we modify the upper bound of Hashin–Shtrikman equation by using cubic spline interpolation to find the additional polynomial terms that are needed to fix the accuracy of the upper bound response. This results in the following equation for the upper bound of the effective permittivity:

10 $$\begin{aligned} \varepsilon _{HS}^U = \varepsilon _x = \varepsilon _y = \varepsilon _a \frac{(2-g)\varepsilon _0 +g\varepsilon _a}{g\varepsilon _0 + (2-g)\varepsilon _a} - 4.573g^3 - 0.5534g^2 + 0.5079g + 0.04922 \end{aligned}$$

For the $\varepsilon _z$ component Eq. (7) is used for all the cases. Figure 4c shows how $\varepsilon _i(i = x,y,z)$ vary as a function of *g* after the introduction of Eq. (10). Furthermore, Fig. 4d compares the analytical response based on our modified Hashin–Shtrikman upper bound of Eq. (10) to the simulated response. It is seen that the agreement between the two is excellent. Specifically, with the addition of the new polynomial terms in Eq. (10), we obtained a maximum relative error that is less than 4%. Also, in Fig. 4f, we compare the response of our TMP honeycomb dielectric structure to the response of a traditional hexagonal honeycomb structure (see Fig. 4e). These results confirm that our TMP dielectric structure behaves like a honeycomb structure. It should be noted that we did not intend to have the two designs exhibit identical responses. Therefore, the slight shift between the reflection coefficients of the two structures is of no significance.

#### TMP absorber

After the EM characterization of our TMP dielectric structure in the previous section, the next steps are to design our TMP absorber and study its performance. The design methodology of our proposed origami absorber follows the design of the Tachi–Miura Polyhedron geometry. An extensive analysis of the Tachi–Miura Polyhedron (TMP) can be found in literature^70,71^.

**Figure 5:**
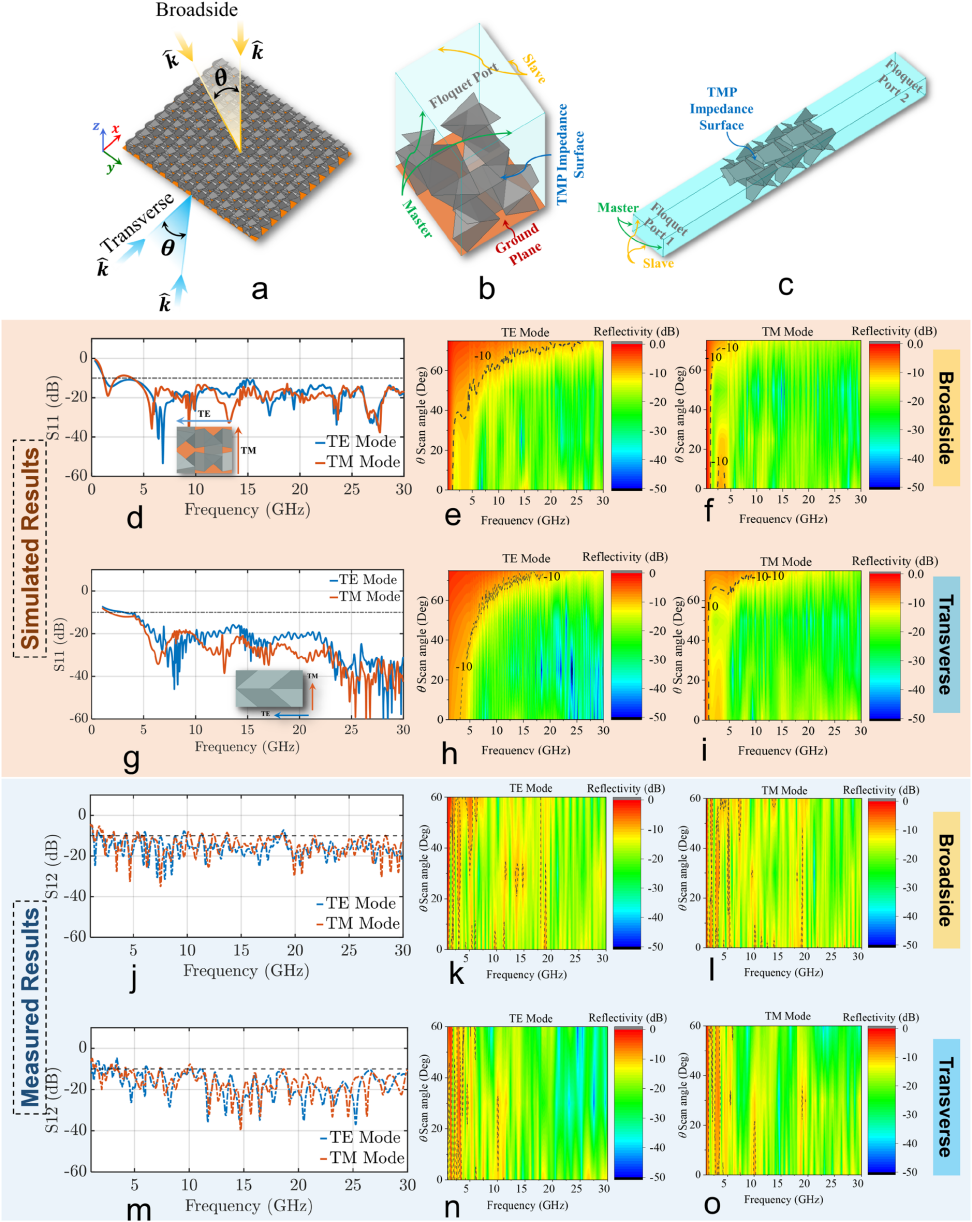
(**a**) A $10\times 10.5$ unit cell array of our proposed TMP absorber. Simulation setup of the TMP absorber unit cell for: (**b**) broadside illumination, and (**c**) transverse illumination. Simulated reflectivity of TMP absorber for TE and TM modes with respect to the structure shown in the inset for: (**d**) broadside illumination and an incidence wave angle of $\theta =0^{\circ}$, and (**g**) transverse illumination and an incidence wave angle of ($\phi =0^{\circ}$ and $\theta =90^{\circ}$). Simulated reflectivity of TMP absorber for different angles of incidence for broadside illumination: (**e**) TE mode, (**f**) TM mode. Simulated reflectivity of TMP absorber for different angles of incidence for transverse illumination: (**h**) TE mode, and (**i**) TM mode. Measured reflectivity of TMP absorber for TE and TM modes for: (**j**) broadside, and (**m**) transverse illumination cases. Measured reflectivity of TMP absorber for different angles of incidence for broadside illumination: (**k**) TE mode, (**l**) TM mode. Measured reflectivity of TMP absorber for different angles of incidence for transverse illumination: (**n**) TE mode, and (**o**) TM mode.

Inspired by honeycomb microwave absorbers^57^, we propose here an origami microwave absorber based on the TMP origami geometry presented in Supplementary Material Section 2.2. Figure 5a shows our $10\times 10.5$ absorber array. Following standard absorber design approaches, we apply a resistive material on the faces of our TMP design and a metallic ground plane at the bottom of our absorber^57^. For all our electromagnetic simulations, we assumed that the dielectric TMP structure is infinitesimally thin (i.e., no dielectric was included in our simulation), and the resistive sheet was simulated with no thickness using an impedance boundary condition. In other words, the entire TMP origami structure was simulated as a resistive sheet with no thickness. The performance of our absorber is analyzed for broadside and transverse illumination using simulations in ANSYS HFSS (see Fig. 5a). A step-by step design process for the 3D structure for our TMP absorber is presented in Figs. S4 and S5 of our Supplementary Material in Section 2.2. The simulation setup for the broadside case uses one unit cell with periodic boundary conditions, and a Floquet port excitation, as shown in Fig. 5b. The simulation setup for the transverse case uses three stacked unit cells with periodic boundary conditions on the periphery of this “extended” unit cell, and a Floquet port excitation, as shown in Fig. 5c. The resistive material is modeled in both cases as a surface impedance of 250 $\Omega {/}\square$. All the geometrical parameters of our unit cell are shown in Fig. 4 in Supplementary Material Section 2.2.

Figures 5d-5i show our simulated results. Specifically, Fig. 5d shows that for the broadside case and an incidence wave angle of $\theta =0^{\circ}$, our TMP origami absorber provides for both TE and TM modes a reflection that is below $-10$ dB (i.e., an absorption that is greater than 90%) in the frequency band between 1.22 GHz and 30 GHz. In addition, Figs. 5e, and 5f illustrate the reflection for different angles of incidence of the TE and TM impinging waves for the broadside illumination, respectively. These results show that our TMP absorber provides consistent absorption of EM waves for incidence angles less than $40^{\circ}$ without sacrificing bandwidth. However, for incidence angles greater than $40^{\circ}$, it is seen that TE impinging waves, are more strongly reflected than TM impinging waves; this reduces the operational bandwidth of the absorber. This can be attributed to the orientation of the EM field components in respect to the aperture of the absorber, and it can be explained using boundary conditions (e.g.,^72^). Specifically, in the case of TE impinging waves, as the angle of incidence $\theta$ increases from $0^{\circ}$ to $90^{\circ}$, the corresponding magnetic field component ($\vec{\mathbf {H}}$) of the incident wave changes its orientation from solely parallel to perfectly perpendicular in respect to the absorber’s aperture. Notably, based on the boundary conditions, for simplicity and without loss of generality, a magnetic field component that is parallel to a perfect conductive surface (PEC) excites equivalent electric currents on this surface ($\hat{n}\times \vec{\mathbf {H}}_{total}=\vec{\mathbf {J}}_s$, where $\hat{n}$ is the unit normal vector on the surface of observation, $\vec{\mathbf {H}}_{total}=\vec{\mathbf {H}}^{inc}+\vec{\mathbf {H}}^{scat}$, and $\vec{\mathbf {J}}_s$ is the electric current that flows on the surface), while a magnetic field component that is perpendicular to a PEC surface is totally reflected ($\hat{n}\cdot \vec{\mathbf {H}}_{total}=0$). However, in the case of TM impinging waves, the corresponding magnetic field component remains parallel to the absorber’s aperture for any angle of incidence, $\theta$. Therefore, TM impinging waves are totally “transformed” to equivalent electric currents on the absorber’s aperture, which dissipates their energy due to the absorptive properties of the structure. On the other hand, TE impinging waves are partially “transformed” to equivalent electric currents and, therefore, partially absorbed, because the corresponding magnetic field component changes its orientation in respect to the absorber’s aperture as the angle of incidence changes. A more detailed analysis of this phenomenon can be found in Section 2.3 of our Supplementary Material. Figure 5g shows that for the transverse case with an incidence wave angle of $\phi =0^{\circ}$ and $\theta =90^{\circ}$, our TMP origami absorber provides for both TE and TM modes a reflection that is below $-10$ dB in the frequency band between 1.2 GHz and 30 GHz (a slightly larger reflection of $-8$ dB is observed for the TE mode in 1.2 GHz to 3 GHz frequency band). In addition, Figs. 5h, and 5i illustrate the reflection for different angles of incidence of the TE and TM impinging waves for the transverse illumination, respectively. These results show that our TMP absorber provides consistent absorption of EM waves for incidence angles less than $40^{\circ}$ without sacrificing bandwidth. However, for incidence angles greater than $40^{\circ}$, it is seen that TE impinging waves are more strongly reflected, thereby reducing the corresponding operational bandwidth of the absorber. Based on the results in Figs. 5d–5i, it can be concluded that for angles less than $40^{\circ}$, our TMP origami absorber provides an ultrawideband performance with a 24.6:1 bandwidth for both the broadside and transverse illumination. Notably, the absorptivity is calculated as^57^:

11 $$\begin{aligned} A = 1 - |S_{11}|^2-|S_{21}|^2 \end{aligned}$$

where, $S_{11}$ is the reflectivity and $S_{21}$ is the transmittivity. Also, due to the conductive ground plane at the back of our absorber, the transmittivity, $S_{21}$, is zero.

### Mechanical analysis

In this section, we study the mechanical characteristics of our TMP honeycomb absorber and compare its performance to the one of the traditional hexagonal honeycomb structure. Our mechanical studies are performed using finite element analysis (FEA).

#### Definition of Tachi–Miura polyhedron

First, we start our analysis by defining the TMP coordinates of the vertices in the flat (i.e., unfolded state) state which are shown in the Supplementary Material Section 2.1. These vertices are then rotated to the desired position ($\gamma _1=80^{\circ}$, which corresponds to $\theta _m=40^{\circ}$) using the known angles between the panels ($\alpha _i$) and the actuation angle ($\gamma _1$). Yasuda and Yang^70^ present the equations for the length (B), width (W), and height (H) of a unit TMP. Modifying these equations to be usable for any $p\times n$ array of unit cells, the equations become:

12 $$\begin{aligned}{}&B=2 m n\sin \bigg [2\tan ^{-1}\big (\tan \alpha \cos \big (\frac{\gamma _1}{2}\big )\big )\bigg ]+\frac{d}{2}\big (n+1\big )\cos \big (\frac{\gamma _1}{2}\big ) \end{aligned}$$

13 $$\begin{aligned}{}&W=2p\bigg [2l+m\cos \big ( 2\tan ^{-1}\big (\cos \big (\frac{\gamma _1}{2}\big )\tan \alpha \big )\big )\bigg ] \end{aligned}$$

**Figure 6:**
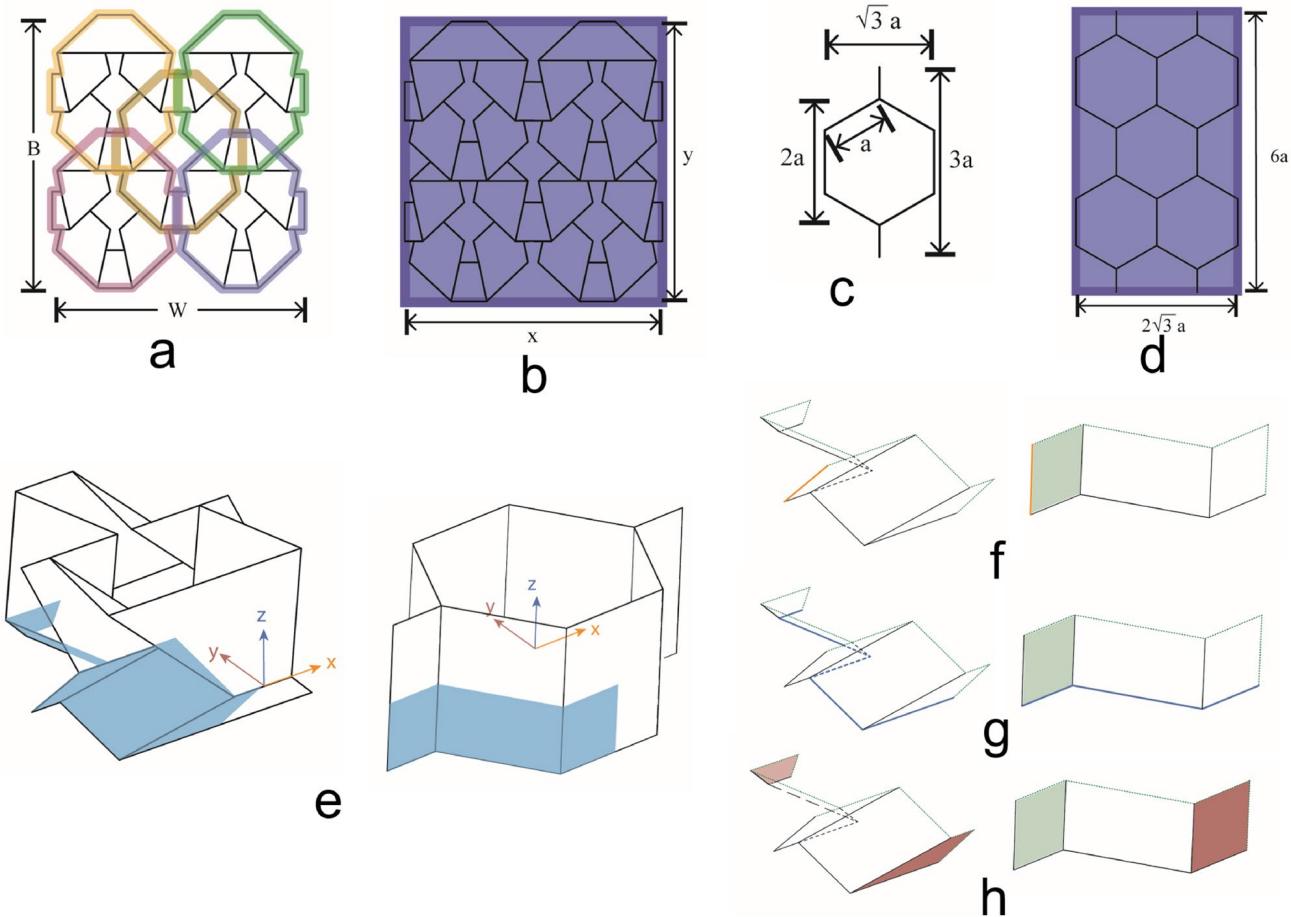
(**a**) A $2 \times 2$ TMP array. Each color shows the outline of a different TMP unit cell. (**b**) The rectangular area that the 5 TMP array occupies is shown by the blue box. (**c**) The parameters of a regular hexagonal honeycomb unit cell. *a* is the side length of the hexagon. All other dimensions are defined in relation to this parameter. (**d**) The area that the 5 hexagon array occupies is shown by the blue box. The width and height dimensions of the box are shown in terms of the sidelength, *a*, of the hexagon. (**e**) The principal directions used for the mechanical analysis of the TMP and Hexagonal Honeycomb. The origin of the TMP is located at the center of the front crease. The origin of the hexagonal honeycomb is located at the center of the hexagonal honeycomb. Subfigures (**f**)–(**h**) show the loading conditions on $\tfrac{1}{4}$ of the TMP and $\tfrac{1}{8}$ of the hexagonal honeycomb. (**f**) The loading conditions in the *x*-direction. The deflection was applied on the orange edge in the positive *x*-direction. (**g**) The loading conditions in the *y*-direction. The deflection was applied on the front face in the positive *y*-direction. A rigid support was placed on the back face, shown in lighter red. (**h**) The loading conditions in the *z*-direction. The deflection was applied on the bottom edge, shown in blue, in the positive *z*-direction. The green dashed lines show where the frictionless supports were applied to each honeycomb structure. The lighter green area shows a frictionless support on the opposite side to the panel view.

Figure 6a shows a $2\times 2$ TMP array with *B* and *W* shown.

#### Comparison of TMP and hexagonal honeycomb arrays

As discussed in “TMP homogenization method” section hexagonal honeycomb arrays have been used for the design of electromagnetic absorbers. A regular (equilateral and equi-angular) hexagonal honeycomb array is compared here with our TMP array. Notably, a regular (equilateral and equi-angular) hexagon is the most common hexagonal honeycomb used. However, a regular hexagon does not exactly match the shape of our TMP, as illustrated in Fig. 6 (also see Supplementary Fig. S7). A regular hexagonal honeycomb has an aspect ratio of $\sqrt{3}$. The aspect ratio of the TMP depends on the desired fold angle in addition to the lengths of the panels. The equation for the aspect ratio (AR) of the TMP is:

14 $$\begin{aligned} AR=\frac{B}{W}=\frac{(n+1)d\cos \big (\frac{\gamma _1}{2}\big ) + 4mn\sin \big [ 2\tan ^{-1}\big (\cos \big (\frac{\gamma _1}{2}\big )\tan \alpha \big ) \big ]}{4p\big (2l+m\cos \big [2\tan ^{-1}\big (\cos \big (\frac{\gamma _1}{2}\big )\tan \alpha \big ) \big ]\big )}. \end{aligned}$$

The aspect ratio for the TMP with the best absorption properties is 1.40. Therefore, a method to determine the corresponding size of a hexagonal honeycomb to TMP is shown. An array of 5 TMP unit cells is created because this is the smallest repeating array, besides the unit cell. An array of 5 TMPs is shown in Fig. 6a, with each unit TMP shown in a different color. Figure 6b shows the rectangular area these 5 TMPs fit inside. The width and height of this array are determined to calculate the rectangular area of the 5-cell TMP. An equivalent hexagonal honeycomb pattern is determined so that it has the same area as the 5-cell TMP array. However, the equivalent hexagonal honeycomb has a different width and height than 5-cell TMP array. Therefore, the following relations can be written:

15 $$\begin{aligned} {\begin{matrix} &{} A_{TMP}=A_{Hex} \\ &{} x_{TMP} \ne x_{Hex} \\ &{} y_{TMP} \ne y_{Hex} \end{matrix}} \end{aligned}$$

Using Eq. (15) and the parameters of a unit hexagonal honeycomb that are shown in Fig. 6c, the side of the hexagonal honeycomb, *a*, can be expressed as follows:

16 $$\begin{aligned} a=\frac{2\bigg [2l+m\cos \big ( 2 \tan ^{-1} \big ( \cos \big (\frac{\gamma _1}{2}\big )\tan \alpha \big )\bigg ]\bigg [3d\cos \big (\frac{\gamma _1}{2}\big )+8m\sin \big (2 \tan ^{-1} \big (\cos \big (\frac{\gamma _1}{2}\big )\tan \alpha \big ) \bigg ]}{12\sqrt{3}}. \end{aligned}$$

The area of the rectangular box and the length of the hexagon sides, *a*, are evaluated and shown in Supplementary Table T4 in our Supplementary Material in Section 2.3, assuming a TMP absorber with the dimensions also shown in Supplementary Table T4.

#### Methodology of determining force-deflection and stress behavior

In this study, we compare the force-deflection behavior and the equivalent stress behavior of the TMP and hexagonal honeycomb structures. A finite element analysis is performed on the TMP and corresponding hexagonal honeycomb for different thicknesses, and in the three principal directions (*x*, *y*, and *z*). The coordinates of the TMP and the hexagonal honeycombs are used to create the zero-thickness models of the TMP and hexagonal honeycomb unit cells. Determination of the TMP coordinates for a specified fold angle ($\gamma _1$) is shown in Supplementary Material Section 2.1. Thickness is added evenly on both sides of the zero thickness model to create a solid model. The sharp edges and corners are filleted with 0.5 mm radii. The TMP model is split into a quarter section based on symmetry. The hexagon is split into an eighth section based on symmetry, as illustrated in Figs. 6e–6h. These models were meshed using solid elements. The mesh is refined along and around the creases until the stress converges to a value that is less than 5% different from the prior stress value.

To determine the force-deflection and stress characteristics, a deflection load is applied to each structure on the edges of the structure for the *x*- and *z*-directions, and on the front faces of the structures in the *y*-direction, as shown in Figs. 6f–6h. A frictionless support is applied to each of the edges in the TMP structure where it was split due to symmetry. Also, a frictionless support is placed on each of the edges and the face [shown in light green in Figs. 6f–6h] where the hexagonal structure was split due to symmetry. The structure is deflected 4 mm in each direction because this is meant to provide support to a structure and bear weight, while not deflecting. Additionally, the TMP will experience self-intersecting deflection at values slightly higher than 4 mm. Figure 6e shows the coordinate system for the TMP and hexagonal honeycomb, and the directions and edges that will be loaded for each principal direction. A single unit cell of each structure is used in FEA, because the unit cell can be added in series or parallel depending on the desired design to find the overall stiffness of the structure.

The TMP and hexagonal honeycomb are analyzed for three different materials using ANSYS Workbench 2021 R2 Mechanical. The properties of these materials are shown in Supplementary Material Table T3. Non-linear geometric effects were enabled. The force to deflect each structure and the corresponding maximum and average Von Mises stresses are determined.

#### Comparison of results

Our numerical analysis is performed for three different materials and three different thicknesses. For brevity, we only present here the data for the aramid paper material, since our results indicate that the mechanical behaviors of the other two materials followed similar trends.

**Figure 7:**
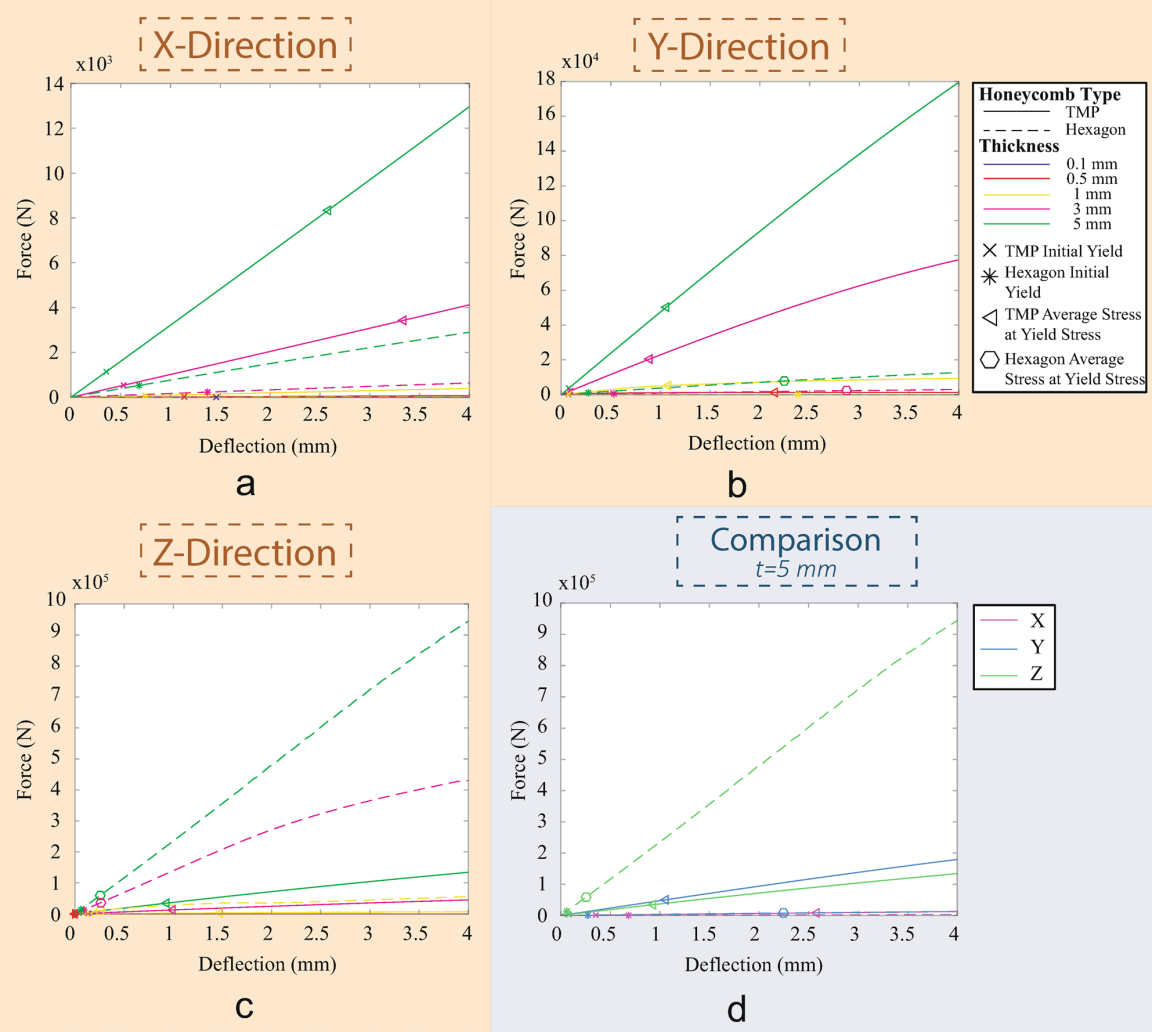
Force-deflection trends for the Tachi–Miura Polyhedron and Hexagonal Honeycombs with Aramid material properties in the *x*, *y*, and *z* directions for the different material thicknesses. (**a**) The *x*-direction force-deflection curves. (**b**) The *y*-direction force-deflection curves. (**c**) The *z*-direction force-deflection curves. (**d**) A comparison of the 5 mm thick TMP and Hexagonal honeycombs’ force-deflection responses in the *x*, *y*, and *z*-directions. The solid lines represent the TMP force-deflection behavior and the dashed lines represent the Hexagonal honeycomb force-deflection behavior. The ‘$\times$’ shows the initial yield point for the TMP. The ‘$*$’ shows the initial yield point for the hexagonal honeycomb. The hexagon shape shows the location where the average stress is at the yield stress for the TMP honeycomb. The triangle shape (‘$\triangleright$’) shows the location where the average stress is at the yield stress for the Hexagonal honeycomb.

Figure 7 shows the comparison between the force-deflection curves of the TMP and the hexagonal honeycomb in the three principal directions. The points shown by an ‘$\times$’ and a ‘$*$’ depict where the initial yielding occurs for the TMP and the hexagonal honeycomb, respectively. For each design, this initial yielding point occurs only at one small point. In a ductile material, yielding will occur at a small point, and the stresses will be redistributed, thereby allowing the structure to continue to bear load without a catastrophic failure. Due to this reason, the point where the average Von Mises stress is equal to the yield stress is also plotted for each design, this gives a closer approximation to where each structure may start to approach failure. These points for the hexagonal honeycomb are shown by hexagonal shapes on the force-deflection curves. The points for the TMP are shown by the triangular shapes on the force-deflection curves.

Figure 7 shows that the TMP is stiffer than the hexagonal honeycomb structure in the *x*-direction and *y*-direction. However, the TMP is not stiffer than the hexagonal honeycomb in the *z*-direction. This is due to the deformation modes of each structure. The hexagonal honeycomb’s deformation mode in the *z*-direction occurs via panel buckling, while the deformation in the TMP is increased due to the horizontal crease stretching around the unit cell. Additionally, the *z*-direction stiffness is much higher than any of the TMP directional stiffnesses. This characteristic is seen in Fig. 7d, where the 5 mm thick TMP and hexagonal honeycomb force-deflection behaviors in the *x*, *y*, and *z*-directions are shown. The stiffness (slope of the force-deflection curve) of the hexagonal honeycomb in the *z*-direction is approximately 5 times the *y*-direction TMP stiffness and about 6 times the *z*-direction TMP stiffness. Based on these results, we can conclude that the mechanical performance of the TMP structure is reasonable. Therefore, the use of the TMP absorber over the hexagonal honeycomb absorber is clearly justified, because our TMP absorber surpasses the performance of all previously presented absorbers (including hexagonal honeycomb absorbers) by exhibiting the widest (to our knowledge) bandwidth ever reported, and exhibits acceptable mechanical performance. The stiffness of the TMP can be increased by using hard stop features. Even though a static structure is proposed here, the origami features could be exploited in the future to create dynamically changing absorbers.

## Methods

**Figure 8:**
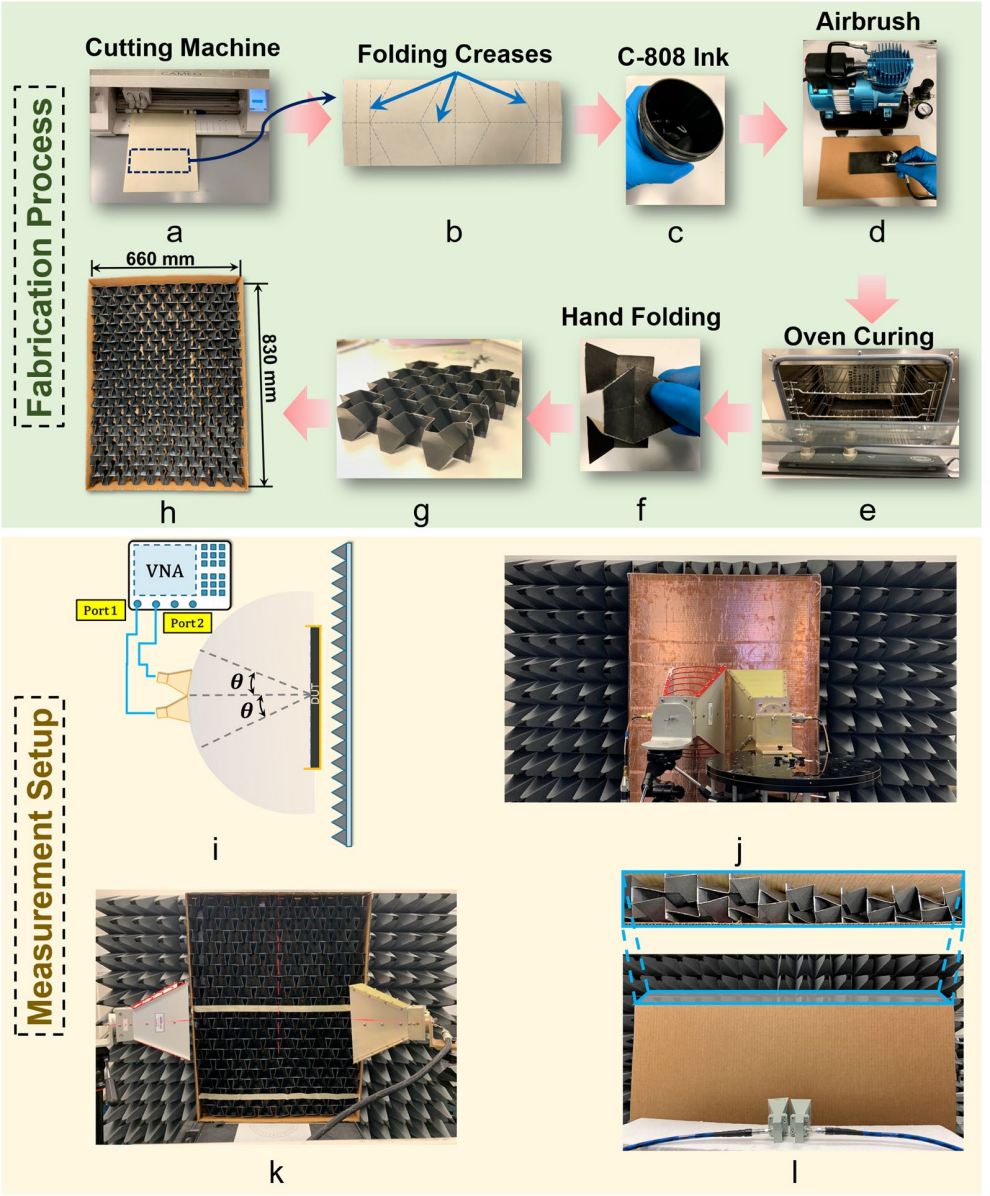
Fabrication process of the TMP absorber: (**a**) cardstock paper is scored using a Silhouette Cameo, (**b**) scored flat front panel, (**c**) C-808 conductive carbon ink, (**d**) spraying ink with airbrush, (**e**) curing in the oven, (**f**) folding flat panel, (**g**) several folded panels joined together, and (**h**) prototype of TMP absorber inside a box frame. Measurement setup of our proposed absorber: (**i**) Schematic of setup. (**j**) Setup used for calibration. (**k**) Setup for broadside illumination with the TE mode at an incident angle of $\theta = 60^{\circ}$. (**l**) Setup for transverse illumination with the TE mode at an incident angle of $\theta = 0^{\circ}$; our absorber is shown in the inset view.

To validate the simulated electromagnetic performance of our TMP absorber, we fabricated a prototype and measured it in our facilities. Figure 8 illustrates our step-by-step fabrication and measurement process.

The fabrication process started by designing the crease pattern in our substrate. A 176 GSM (0.198 mm) cardstock paper was used as our origami substrate. A Silhouette Cameo 3 cutting machine was used to score the origami pattern as shown in Fig. 8a. Figure 8b shows the scored flat front panel with the corresponding creases. We ink-sprayed our flat front panel with a conductive ink to form the desired resistive layer. We used a water-based highly conductive *C*-808 carbon ink^73^ as shown in Fig. 8c. A standard airbrush machine was used to spray the carbon ink on both sides of the scored substrate. As shown in Fig. 8d, the carbon ink was sprayed on our absorber when it still at its flat state. We optimized the number of sprays and the ink deposition level to create a resistive sheet with approximately 250 $\Omega$ surface impedance. We cured the sprayed panel in the oven for 10 min at $100^{\circ}$ Celsius, shown in Fig. 8e. Then, we hand-fold the panel to the specific folded state of $\theta _m=40^{\circ}$. The folded panel is shown in Fig. 8f. We repeated all these steps, to create the required number of folded panels. Then, we joined all the unit cells together to form the complete TMP structure, as shown in Fig. 8g. Our final TMP absorber prototype consists of $10\times 10.5$ unit cells, and has dimensions $660\times 830\,\mathrm{mm}^2$, as shown in Fig. 8h. To maintain the desired folding state of $\theta _m = 40^{\circ}$, we made a cardboard box and used it to frame our absorber, as shown in Fig. 8h.

To measure the electromagnetic response of our TMP absorber, we created the free-space measurement setup shown in Fig. 8i. To measure the reflectivity performance of our TMP absorber between 1 and 30 GHz, we used three pairs of horn antennas to cover the desired frequency range, and two network analyzers. The horn antennas and network analyzer specifications are given in Supplementary Material Table T5.

To calibrate our measurement setup, we used a ground plane (i.e., conductive sheet) as a perfect reflector, as shown in Fig. 8j. Also, in our measurement setup, we placed a wall with commercially available pyramidal absorbers behind our ground plane to eliminate any undesired reflections. For the measurements we conducted at each of our three frequency bands (see Supplementary Material Table T5), we set a $0.5\lambda$ distance between the absorber and the horn antennas, where $\lambda$ represents the wavelength at the lowest operating frequency of each band (i.e., 1 GHz, 6 GHz, 18 GHz). The incident angle of the impinging EM waves was varied by azimuthally rotating the horn antennas, as shown in Fig. 8i. Notably, we measured both the broadside and transverse illumination cases using the same measurement setup. Figure 8k shows the broadside TE mode illumination measurement setup of our TMP absorber for an incidence angle of $60^{\circ}$. The TM mode responses are measured by rotating our absorber by $90^{\circ}$. Also, to conduct the transverse case measurements, we modified our absorber by stacking several unit cell columns on top of each other. Specifically, we stacked 8 columns to form the desired aperture for the transverse illumination. Figure 8l shows our measurement setup of the transverse TE mode illumination case for an incidence angle of $0^{\circ}$. As seen, our absorber is placed inside a cardboard box.

Figure 5j shows our measurements for the broadside case for both TE and TM polarized waves. These results show that our TMP absorber maintains a reflection less than $-10$ dB in most of the frequency band between 1.27 and 30 GHz, and it exhibits a slightly larger reflection of $-8$ dB only in few narrow bands in this frequency range. Also, our measurements for different angles of incidence are shown in Figs. 5k, 5l. Our measured results agree very well with our simulations (shown in Figs. 5e, and 5f), except with the appearance of few frequencies in the band between 1.27 GHz and 30 GHz, where the reflection is slightly higher, i.e., $-8$ dB. Figures 5m–5o show our measurements for the transverse case for both TE and TM polarized waves. We see again that our measurements agree very well with our simulations (shown in Figs. 5g–5i), while some discrepancies between the two appear at lower frequencies.

In summary, the excellent agreement between our measurements and simulations, validates the electromagnetic performance of our absorber, which is the first (to our knowledge) to achieve a 24.6:1 bandwidth. There were some slight discrepancies between the measurements and simulations at certain frequencies, which can be attributed to the following: (a) inhomogeneous ink deposition during our fabrication, since we sprayed the conductive ink by hand (which can add some variation in the surface resistance value of all the pieces we fabricated), (b) joining misalignments and inhomogeneous deformations of our paper prototype that might have inadvertently occured during our assembly process, and (c) folding angle deviations from the exact desired angle, since we folded all the unit cells by hand.

## Discussions

A new ultra-wideband absorber, which provides 4.5 times greater bandwidth than state-of-the-art honeycomb absorbers, was presented here. Specifically, we used a TMP origami pattern to create the first honeycomb origami absorber that achieves a 24.6:1 bandwidth with absorptivity of more than 90%. Namely, by utilizing the origami’s spatial characteristics (in other words, the TMP’s physical deformation) we enhanced the electromagnetic performance of our absorber. We explained the ultrawideband behavior that we achieve by utilizing theoretical models of multi-layer inhomogeneous media to describe the transmission-reflection of propagating waves through them. In addition, we introduced a homogenization approach for our TMP dielectric structure, which enabled the accurate and fast analysis of our absorber. We performed a rigorous study of the EM and mechanical performance of our absorber using simulation tools. Our electromagnetic simulation results were validated by fabricating and measuring a prototype of our absorber. Notably, our simulations agreed very well with our measurements in the entire bandwidth of operation, and only few discrepancies were observed. These discrepancies are due to fabrication imperfections. Also, because our absorber can be folded from a flat sheet (like any other origami design) it is inexpensive with minimal fabrication and assembly cost. Notably, this work clearly demonstrates that origami can be used in electromagnetics to derive new designs that provide unprecedented EM performance, which greatly surpasses the performance of traditional designs. This is an important finding that can be explored further to develop novel electromagnetic systems.

## Supplementary Information

Supplementary Information.

## Data Availability

All data generated or analysed during this study are included in this published article [and its supplementary information files].
