# Supplementary material for: An ultra-wideband origami microwave absorber
Source: Sci Rep. 2022 Aug 4;12:13449. doi: 10.1038/s41598-022-17648-4 (PMC9352738; doi:10.1038/s41598-022-17648-4)
Supplement: Supplementary file 1 — Supplementary Information. [file 41598_2022_17648_MOESM1_ESM.pdf]

1 Analytical Model

In this section, we theoretically explain and prove the ultra-wideband response of the proposed TMP absorber. The question that needs to be answered is: what is the main characteristic that introduces this behavior? Our hypothesis is that the ultra-wideband performance is a result of the periodic placement of dielectric and air slabs, which is not done in traditional homogeneous absorbers. To prove our assumption we will compare the achievable BW of an inhomogeneous absorber with the achievable bandwidth of a homogeneous one. In both cases, the absorbers are coated with highly resistive ink, which is represented here as a surface impedance Z_{ink}. Also, both absorbers are backed by a perfectly conductive layer. To compare the achievable BWs, we use Rozanov's work,[1] where he showed that the operating BW of a radar absorber is bounded by the following equation:

$$ln|\Gamma|(\lambda_{max} - \lambda_{min}) < 2\pi^2 \sum_i \mu_{s,i} d_i \tag{1}$$

where Γ is the reflection coefficient in the operating frequency range f_{min} to f_{max} corresponding to wavelength range λ_{max} to λ_{min}, $\mu_{s,i}$ is the static permeability (in our case is always 1), and d_i is the thickness of the ith layer of the multi-layer slab. From equation (1) we see that the bandwidth corresponding to λ_{max} to λ_{min} is inversely proportional to the reflection coefficient. Therefore, if we show that the reflection coefficient of the inhomogeneous absorber is smaller than the reflection coefficient of the homogeneous one, correspondingly the bandwidth of the inhomogeneous absorber will be larger than the bandwidth of the homogeneous one. In what follows, we present an analytical approach based on transmission-reflection theory,[2] which proves that the inhomegenous multi-layered dielectric structures that are coated with impedance layers exhibit larger bandwidth than homogeneous dielectrics that are coated with an impedance layer.

1.1 Transmission-Reflection Theory

First, we consider the traditional case of two homogeneous domains (Domain I and II) backed by a perfect electric conducting (PEC) plate, shown in Fig. S1a. Using transmission-reflection theory[2] at the interface of the two domains, we find the electric field amplitude of impinging ($\overrightarrow{E}^i$), reflected ($\overleftarrow{E}^r$), and transmitted ($\overrightarrow{E}^t$) waves as:

$$\overrightarrow{E}^i_I = \hat{x}E_0 e^{-j\beta_I Z} \tag{2}$$

$$\overleftarrow{E}^r_I = \hat{x}\Gamma^b E_0 e^{j\beta_I Z} \tag{3}$$

$$\overrightarrow{E}^r_{II} = -\hat{x}\Gamma^b E_0 e^{j\beta_{II} Z} \tag{4}$$

$$\overleftarrow{E}^t_I = -\hat{x}T^b E_0 e^{-j\beta_{II} d} \tag{5}$$

$$\overrightarrow{E}^t_{II} = \hat{x}T^b E_0 e^{-j\beta_I Z} \tag{6}$$

Note that the direction of the vector over the electric field denotes the direction of propagation, which is towards the $+\hat{z}$ for the right vector, and towards the $-\hat{z}$ for the left vector, respectively. Here, Γ^b and T^b are the reflection and transmission coefficients, respectively, at the interface of the two domains. Magnetic field equations can be similarly written and they are omitted here for reasons of brevity. By applying the appropriate boundary condition, we obtain the following equations:

$$1 + \Gamma^b = T^b \tag{7}$$

$$\frac{1}{\eta_I}(1 - \Gamma^b) = \frac{1}{\eta_{II}} T^b \tag{8}$$

Solving equations (7) and (8), gives us

$$\Gamma^b = \frac{\eta_{II} - \eta_I}{\eta_{II} + \eta_I} = \frac{E^r}{E^i} \tag{9}$$

$$T^b = \frac{2\eta_{II}}{\eta_I + \eta_{II}} = 1 + \Gamma^b = \frac{E^t}{E^i} \tag{10}$$

Next, we consider the case of Fig. S1b, where we define domain I as air, domain II as dielectric and an infinitesimally thin layer of impedance Z in between. By applying conditions in equation (9) and equation (10), we have:

$$\Gamma^{I,Z} = \frac{Z - \eta_0}{Z + \eta_0} \tag{11}$$

$$T^{I,Z} = \frac{2Z}{Z + \eta_0} \tag{12}$$

$$\Gamma^{Z,I} = \frac{\eta_0 - Z}{\eta_0 + Z} \tag{13}$$

$$T^{Z,I} = \frac{2\eta_0}{\eta_0 + Z} \tag{14}$$

$$\Gamma^{II,Z} = \frac{Z - \eta}{Z + \eta} \tag{15}$$

$$T^{II,Z} = \frac{2Z}{Z + \eta} \tag{16}$$

$$\Gamma^{Z,II} = \frac{\eta - Z}{\eta + Z} \tag{17}$$

$$T^{Z,II} = \frac{2\eta}{\eta + Z} \tag{18}$$

Here, the notation k,l of the the superscripts of Γ and T is used to define the direction of arrival of the impinging wave. For example, when it is $k,l = I,Z$, impinging wave travelling in domain I impinges on the Z impedance layer. Symbols η_0 and η denote the intrinsic impedances of domains I and II, respectively, and Z is the characteristic impedance of the impedance layer. The thickness, t, of the impedance layer is assumed to be infinitesimal ($t \to 0$), while the thickness of domain II is d. By using equations (2)-(6) and equations (11)-(18) we can write the electric field of each domain as:

<u>Domain I</u>:

$$\overleftarrow{E}_I^r = \Gamma^{I,Z} \cdot \overrightarrow{E}^i \tag{19}$$

$$\overleftarrow{E}_I^t = T^{Z,I} \cdot \overleftarrow{E}^Z \tag{20}$$

$$\overleftarrow{E}^r = \overleftarrow{E}_I^r + \overleftarrow{E}_I^t \tag{21}$$

Domain Z:

$$\overleftarrow{E}_Z^r = \Gamma^{Z,II} \cdot \overrightarrow{E}_Z^t e^{-j\beta_z t} \tag{22}$$

$$\overrightarrow{E}_Z^t = T^{I,Z} \cdot \overrightarrow{E}^i e^{-j\beta_z t} \tag{23}$$

$$\overleftarrow{E}_Z^t = T^{II,Z} \cdot \overleftarrow{E}^{II} e^{-j\beta_z t} \tag{24}$$

$$\overleftarrow{E}^Z = \overleftarrow{E}_Z^r + \overleftarrow{E}_Z^t \tag{25}$$

Domain II:

$$\overleftarrow{E}_{II}^r = -\overrightarrow{E}_{II}^t \cdot e^{-j\beta d} \tag{26}$$

$$\overrightarrow{E}_{II}^t = T^{Z,II} \cdot \overrightarrow{E}_Z^t e^{-j\beta d} \tag{27}$$

$$\overleftarrow{E}^{II} = \overleftarrow{E}_{II}^r \tag{28}$$

To find the reflected E-field component in domain I, substitute in equation (21) the corresponding field components using equations (19), (20), (25), and (28):

$$\overleftarrow{E}^r = \Gamma^{I,Z} \cdot \overrightarrow{E}^i + (\Gamma^{Z,II} \cdot T^{I,Z} \cdot \overrightarrow{E}^i \cdot e^{-2j\beta_Z t} - T^{II,Z} \cdot T^{Z,II} \cdot T^{I,Z} \cdot \overrightarrow{E}^i \cdot e^{-2j\beta d} \cdot e^{-j\beta_z t}) T^{Z,I} \tag{29}$$

Finally, using equation (29) we calculate the total reflection as,

$$\overleftarrow{E}^r = \Gamma^{total} \cdot \overrightarrow{E}^i \tag{30}$$

where,

$$\Gamma^{total} = \Gamma^{I,Z} + T^{Z,I} \cdot \Gamma^{Z,II} \cdot T^{I,Z} \cdot e^{-2j\beta_Z t} - T^{Z,I} \cdot T^{II,Z} \cdot T^{Z,II} \cdot T^{I,Z} \cdot e^{-2j\beta d} \cdot e^{-j\beta_z t} \tag{31}$$

It is clearly evident in equation (31) that the total reflection coefficient depends on the reflection (Γ) and transmission (T) coefficients of the interface, the thickness of the dielectric slab (d) and the thickness (t) of the impedance layer.

To examine the multi-layer case, we consider two layers of dielectric that are separated by an air layer, shown in Fig. S1b. Both dielectric layers are coated with the same impedance layer. Notably, the total thickness of the two dielectric layers with the air layer between them is equal to the thickness of the single dielectric layer of Fig. S1a. Therefore, it is $d_1 + d_2 + d_3 = d$. The reflection and transmission coefficients of the first two domains separated by the impedance boundary layer are given by equations (11)-(18), while the corresponding coefficients for the rest of the layers are written as:

$$\Gamma^{II,III} = \frac{\eta_0 - \eta}{\eta_0 + \eta} \tag{32}$$

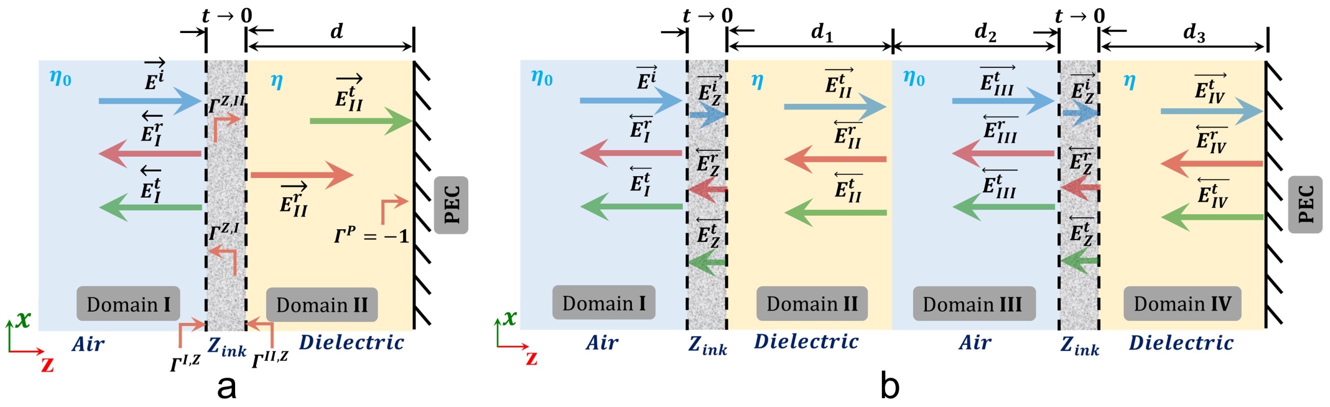

Figure S 1. Schematic diagram of media coated with a resistive layer of Z_{ink} impedance. (a) Homogeneous medium with one dielectric layer, and (b) inhomogeneous medium with two dielectric layers and one air layer.

$$T^{II,III} = \frac{2\eta_0}{\eta_0 + \eta} \tag{33}$$

$$\Gamma^{III,II} = \frac{\eta - \eta_0}{\eta + \eta_0} \tag{34}$$

$$T^{III,II} = \frac{2\eta}{\eta + \eta_0} \tag{35}$$

$$\Gamma^{III,Z} = \Gamma^{I,Z} \tag{36}$$

$$T^{III,Z} = T^{I,Z} \tag{37}$$

$$\Gamma^{Z,III} = \Gamma^{Z,I} \tag{38}$$

$$T^{Z,III} = T^{Z,I} \tag{39}$$

$$\Gamma^{IV,Z} = \Gamma^{II,Z} \tag{40}$$

$$T^{IV,Z} = T^{II,Z} \tag{41}$$

$$\Gamma^{Z,IV} = \Gamma^{Z,II} \tag{42}$$

$$T^{Z,IV} = T^{Z,II} \tag{43}$$

Therefore, the corresponding electric field components in each domain are written as follows:
<u>Domain I</u>:

$$\overleftarrow{E}^{r}_{I} = \Gamma^{I,Z} \cdot \overrightarrow{E}^{i} \tag{44}$$

$$\overleftarrow{E}^{t}_{I} = T^{Z,I} \cdot \overleftarrow{E}^{Z_1} \tag{45}$$

$$\overleftarrow{E}^{r} = \overleftarrow{E}^{r}_{I} + \overleftarrow{E}^{t}_{I} \tag{46}$$

<u>Domain Z_1</u>:

$$\overleftarrow{E}^{r}_{Z_1} = \Gamma^{Z,II} \cdot \overrightarrow{E}^{t}_{Z_1} e^{-j\beta_Z t} \tag{47}$$

$$\overrightarrow{E}^{t}_{Z_1} = T^{I,Z} \cdot \overrightarrow{E}^{i} e^{-j\beta_Z t} \tag{48}$$

$$\overleftarrow{E}^{t}_{Z_1} = T^{II,Z} \cdot \overleftarrow{E}^{II} e^{-j\beta_Z t} \tag{49}$$

$$\overleftarrow{E}^{Z_1} = \overleftarrow{E}^{r}_{Z_1} + \overleftarrow{E}^{t}_{Z_1} \tag{50}$$

<u>Domain II</u>:

$$\overleftarrow{E}^{r}_{II} = \Gamma^{II,III} \cdot \overrightarrow{E}^{t}_{II} e^{-j\beta d} \tag{51}$$

$$\overrightarrow{E}^{t}_{II} = T^{Z,II} \cdot \overrightarrow{E}^{t}_{Z_1} e^{-j\beta d} \tag{52}$$

$$\overleftarrow{E}^{t}_{II} = T^{III,II} \cdot \overleftarrow{E}^{II} e^{-j\beta d} \tag{53}$$

$$\overleftarrow{E}^{II} = \overleftarrow{E}^{r}_{II} + \overleftarrow{E}^{t}_{II} \tag{54}$$

<u>Domain III</u>:

$$\overleftarrow{E}^{r}_{III} = \Gamma^{III,Z} \cdot \overrightarrow{E}^{t}_{III} e^{-j\beta_0 d} \tag{55}$$

$$\overrightarrow{E}^{t}_{III} = T^{II,III} \cdot \overrightarrow{E}^{t}_{II} e^{-j\beta_0 d} \tag{56}$$

$$\overleftarrow{E}^{t}_{III} = T^{Z,III} \cdot \overleftarrow{E}^{Z_2} e^{-j\beta_0 d} \tag{57}$$

$$\overleftarrow{E}^{III} = \overleftarrow{E}^{r}_{III} + \overleftarrow{E}^{t}_{III} \tag{58}$$

<u>Domain Z_2</u>:

$$\overleftarrow{E}^{r}_{Z_2} = \Gamma^{Z,IV} \cdot \overrightarrow{E}^{t}_{Z_2} e^{-j\beta_Z t} \tag{59}$$

$$\overrightarrow{E}^{t}_{Z_2} = T^{III,Z} \cdot \overrightarrow{E}^{t}_{III} e^{-j\beta_Z t} \tag{60}$$

$$\overleftarrow{E}^{t}_{Z_2} = T^{IV,Z} \cdot \overleftarrow{E}^{IV} e^{-j\beta_Z t} \tag{61}$$

$$\overleftarrow{E}^{Z_2} = \overleftarrow{E}^{r}_{Z_2} + \overleftarrow{E}^{t}_{Z_2} \tag{62}$$

<u>Domain IV</u>:

$$\overleftarrow{E}^{r}_{IV} = -\overrightarrow{E}^{t}_{IV} \tag{63}$$

$$\overrightarrow{E}^{t}_{IV} = T^{Z,IV} \cdot \overrightarrow{E}^{t}_{Z_2} e^{-j\beta d} \tag{64}$$

$$\overleftarrow{E}^{t}_{IV} = \overleftarrow{E}^{r}_{IV} \tag{65}$$

$$\overleftarrow{E}^{IV} = \overleftarrow{E}^{r}_{IV} = \overleftarrow{E}^{t}_{IV} \tag{66}$$

To find the reflected E-field component in domain I we use equation (46):

$$\overleftarrow{E}^{r} = \Gamma^{I,Z} \overrightarrow{E}^{i} + T^{Z,I} \overleftarrow{E}^{Z_1} \tag{67}$$

where,

$$\overleftarrow{E}^{Z_1} = \Gamma^{Z,II} \cdot T^{I,Z} \cdot \overrightarrow{E}^{i} e^{-2j\beta_Z t} + T^{III,II} \cdot \overleftarrow{E}^{II} e^{-j\beta d} \tag{68}$$

$$\overleftarrow{E}^{II} = \Gamma^{II,III} \cdot T^{Z,II} \cdot T^{I,Z} \overrightarrow{E}^{i} e^{-j\beta_Z t} e^{-2j\beta d} + T^{III,II} \cdot \overleftarrow{E}^{III} e^{-j\beta d} \tag{69}$$

$$\overleftarrow{E}^{III} = \Gamma^{III,Z} \cdot T^{II,III} \cdot T^{Z,II} \cdot T^{I,Z} \overrightarrow{E}^{i} e^{-j\beta_Z t} e^{-j\beta d} \cdot e^{-2j\beta_0 d} + T^{Z,III} \cdot \overleftarrow{E}^{Z_2} e^{-j\beta_0 d} \tag{70}$$

$$\overleftarrow{E}^{Z_2} = \Gamma^{Z,IV} \cdot T^{III,Z} \cdot T^{II,III} \cdot T^{Z,II} \cdot T^{I,Z} \overrightarrow{E}^{i} e^{-3j\beta_Z t} e^{-j\beta d} \cdot e^{-j\beta_0 d} + T^{IV,Z} \cdot \overleftarrow{E}^{IV} \tag{71}$$

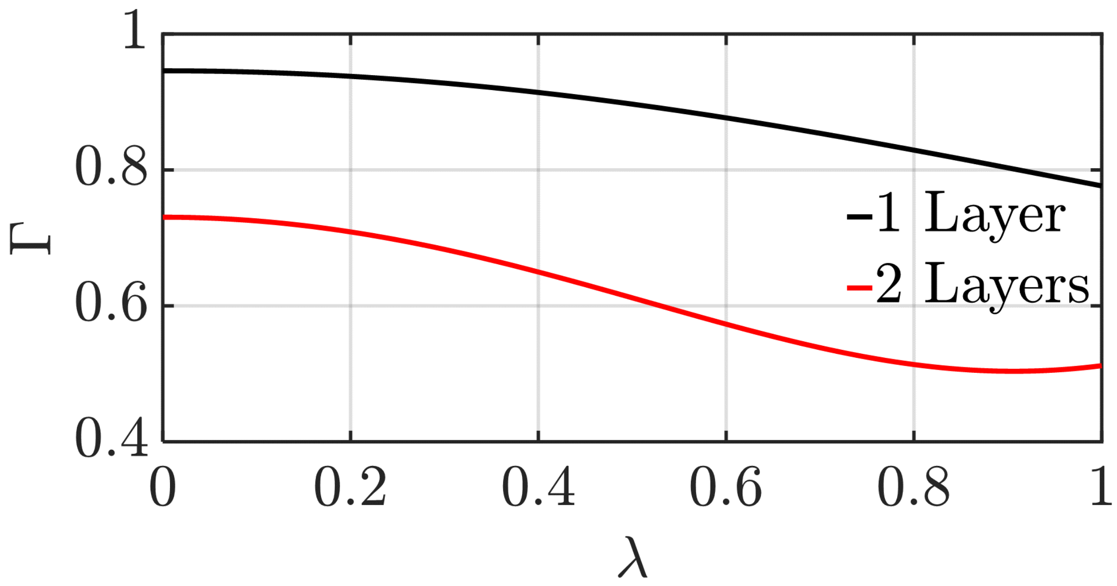

Figure S 2. Analytically calculated reflection coefficient of the 1-layer homogeneous and 2-layer inhomogeneous media.

$$\overleftarrow{E}^{IV} = T^{Z,IV} \cdot T^{III,Z} \cdot T^{II,III} \cdot T^{Z,II} \cdot T^{I,Z} \overleftarrow{E}^{i} e^{-2j\beta d} e^{-2j\beta_Z t} e^{-j\beta_0 d} \tag{72}$$

By substituting equations (68)-(72) into equation (67), we can express $\overleftarrow{E}^{r}$ in terms of the incident wave component, $\overrightarrow{E}^{i}$. Consequently, the total reflection can be calculated using equation (30). Figure S2 shows the reflection coefficients for both the inhomogeneous and homogeneous absorbers. It is seen that the reflection coefficient of the inhomogeneous medium is lower than the reflection coefficient of the homogeneous medium in the entire range of frequencies. Therefore, based on the relationship (which was explained above) between the reflection coefficient and the bandwidth in equation (1), we expect that an absorber that has the same properties with the inhomogeneous medium will exhibit wider bandwidth than an absorber that has the same properties with the homogeneous medium.

2 Tachi-Miura Polyhedron (TMP) Absorber

An extensive analysis of the Tachi-Miura Polyhedron (TMP) can be found in literature.[3,4] For the readers' convenience, we concisely present here the TMP origami design process.

2.1 Definition of Tachi-Miura Polyhedron

The Tachi-Miura Polyhedron (TMP) is defined by defining the coordinates in each panel in each panel's local coordinate frame as shown in Fig. S3. The coordinates of each panel around the first vertex are as follows:

$$
\begin{aligned}
&d_{11} = [0,0,0] \\
&d_{12} = [d\cot\alpha, d, 0] \\
&d_{13} = [2l, d, 0] \\
&d_{14} = [2l + d\cot\alpha, 0, 0] \\
\\
&d_{21} = [0,0,0] \\
&d_{22} = [m\cos\alpha, m\sin\alpha, 0] \\
&d_{23} = [d\csc\alpha + m\cos\alpha, m\sin\alpha, 0] \\
&d_{24} = [d\csc\alpha, 0, 0] \\
\\
&d_{31} = [0,0,0] \\
&d_{32} = [d\cot\alpha, d, 0] \\
&d_{33} = [d\cot\alpha + m, d, 0] \\
&d_{34} = [m, 0, 0] \\
\\
&d_{41} = [0,0,0] \\
&d_{42} = [-(l - d\cot\alpha)\cos\alpha, (l - d\cot\alpha)\sin\alpha, 0] \\
&d_{43} = [d\csc\alpha - (l + \cot\alpha)\cos\alpha, (l + \cot\alpha)\sin\alpha] \\
&d_{44} = [d\csc\alpha, 0, 0]
\end{aligned}
\tag{73}
$$

Figure S3 shows how the initial vertices were determined. The panel coordinates are then rotated to the desired position in the global coordinate frame ($\gamma_1 = 80^o$) using the known angles between the panels (α_i), and the actuation angle (γ_1)

$$
\begin{aligned}
&R_1 = R_x(\pi - \alpha)R_z(\gamma_1) \\
&R_2 = R_1 R_x(\alpha)R_z(\gamma_2) \\
&R_3 = R_2 R_x(\alpha)R_z(\gamma_3) \\
&R_4 = R_3 R_x(\pi - \alpha)R_z(\gamma_4)
\end{aligned}
\tag{74}
$$

where R_i is the rotation matrix of the ith panel and R_x and R_z are the rotation matrices about x and z, respectively. Also γ_1 - γ_4 can be found from γ_1 using well-known relations. This process is repeated for the second vertex shown with an added displacement from the first vertex, and for the vertices on the back side of the pattern.

2.2 TMP - 3D Electromagnetic CAD Model

To model and study our TMP absorber ANSYS HFSS is used. Figures S4 and S5 show the steps that we follow to construct the TMP structure in ANSYS HFSS using its $3D$ modeler.

Specifically, Fig. S4 shows in detail how a flat (i.e., unfolded) panel (that is the main building block of the TMP unit cell) folds to the final folded panel. Figure S5, in turn, shows all the folding steps of the TMP unit cell geometry. Specifically, first, Fig. S5a presents the unfolded (i.e., flat) origami pattern of the front panel of the TMP unit cell with its corresponding geometrical parameters of length (l and m), width (d), and internal angle (α). Second, the origami pattern is folded as shown in Fig. S5b, and the geometric parameters B, W, H of the folded shape are calculated as:[3,4]

$$B = 2m\sin\theta_g + d\cos\theta_m \tag{75}$$

$$W = 2l + \frac{d}{\tan\alpha} + 2m\cos\theta_g \tag{76}$$

$$H = 2d\sin\theta_m \tag{77}$$

$$2l - d\cot\alpha + 2m\cos 2\alpha > 0 \tag{78}$$

where θ_m denotes the folding angle of TMP pattern. Notably, while $\theta_m \in [0^o, 90^o]$, l, m, d, and α should satisfy equation (78).[3] Consequently, if we combine equations (76) and (78), it can be found that $\theta_g \in [0, 2\alpha]$. For our design we specify the values of l, m, d, and α as shown in Table T1. Then, parameter θ_m is calculated by optimizing the performance of our TMP absorber in terms of bandwidth using full-wave simulations. As shown in Fig. S6 the optimal value of θ_m is found to be 40° (for this value of θ_m, θ_g is 99.85^o), while for greater angles of θ_m, the performance of our absorber degrades significantly. For example, in the entire bandwidth of operation, an average of 20 dB reduction in absorption is observed as θ_m increases from 40° to 80°. Notably, the value of θ_m is not changed throughout our analysis as we assume that our design operates only at one fixed state of the TMP origami pattern. The rest dependent geometric parameters of our design, B, W and H are calculated using equations (75)-(77), and they are shown in Table T1. Also, it should be pointed out that θ_m cannot be less than 36° for the specific geometry of our design, as this will cause the intermediate walls of the TMP structure to collide. Third, we complete the TMP unit cell by doing the following: (a) we mirror the folded pattern of Fig. S5b (see result in Fig. S5c), and (b) we copy and translate the geometries in Fig. S5c as shown in Fig. S5d. The top view and perspective views of our complete TMP unit cell are shown in Figs. S5e and S5f, respectively. In addition, Fig. S5f depicts the dimensions X, Y and H of the TMP unit cell, and these are expressed in terms of the upper and lower operating frequency wavelengths in Table T2.

2.3 TMP - Absorptivity Analysis

As discussed in Section 2.1.3 of the main manuscript, our TMP absorber provides an ultrawideband performance with a 24.6 : 1 bandwidth for both the broadside and transverse illumination and for incident waves with angles of incidence that are less than 40°. Specifically, our simulation results showed that for both the broadside ($\phi = 0^o$, $\theta = 0^o$) and transverse ($\phi = 0^o$, $\theta = 90^o$) case, and incidence waves with angles of incidence $\theta = 0^o$ and $\theta = 90^o$, respectively, the TMP origami absorber provides for both TE and TM modes a reflection that is below 10 dB in the frequency band between 1.22 GHz and 30 GHz (see Figs. 5d and 5g in the main manuscript). In addition, Figs. 5e, 5f and 5h, 5i of the main manuscript illustrated consistent absorption of EM waves for incidence angles less than 40° and without sacrificing bandwidth for both the TE and TM impinging waves. Notably, for incidence angles greater than 40°, we showed that TE impinging waves are more strongly reflected, thereby, reducing the corresponding operational bandwidth of the absorber. To explain this behavior, we use the boundary conditions of electromagnetic waves. Let us take the case of an impinging TE wave as shown in Fig. S7a. For reasons of simplicity and without loss of generality, we make the following assumptions: a) the electric field component of the incident wave, $\vec{E}^{inc}$, is parallel to the $\hat{y}$-direction (the local $x'y'z'$ coordinate system shown in Fig. S7a is parallel to the global xyz coordinate system), b) we model a plane wave and, therefore, the electric ($\vec{E}^{inc}$) and magnetic ($\vec{H}^{inc}$) field components are always perpendicular to each other and they are also perpendicular to the direction of propagation $\hat{k}$, and c) the incident wave (see the direction of propagation $\hat{k}$ of the incident wave in Fig. S7a) belongs to the cut plane $\phi = 0^o$ and forms an angle θ with axis-z; notably, all our studies in Section 2.1.3 of the main manuscript follow the same assumptions. Figure S7b shows equivalently the case of a TM incident wave, where now the magnetic field component ($\vec{H}^{inc}$) is taken to be parallel to the $\hat{y}$-direction. What is important to note here is the orientation of the magnetic field component for both the TE and TM impinging waves, as the angle of incidence θ changes. Namely, as we can see from Fig. S7a, as the angle θ increases from 0° to 90°, the magnetic field component of the incident wave changes its orientation from solely parallel to nearly perpendicular in respect to the absorber's aperture. On the other hand, in the TM impinging wave case (see Fig. S7b) the corresponding magnetic field component remains parallel for any angle of incidence, θ. Next, let a plane wave with angle of incidence θ impinge on a perfect electric conductive (PEC) plane defined by its unit normal vector $\hat{n}$. The boundary conditions are,[5]:

$$\hat{n} \times \vec{H}_{total} = \vec{J}_s \tag{79}$$

$$\hat{n} \times \vec{E}_{total} = 0 \tag{80}$$

$$\hat{n} \cdot \mu \vec{H}_{total} = 0 \tag{81}$$

$$\hat{n} \cdot \varepsilon \vec{E}_{total} = \rho_s \tag{82}$$

Table T 1. TMP unit cell parameters for different folding angles (θ_m).

$Parameters$	$\theta_m = 40^o$	$\theta_m = 60^o$	$\theta_m = 80^o$
α	57.2^o	57.2^o	57.2^o
$l(mm)$	20	20	20
$m(mm)$	40	40	40
$d(mm)$	33.62	33.62	33.62
$H(mm)$	43.22	58.24	66.22
$B(mm)$	104.68	94.30	46.03
$W(mm)$	47.98	81.55	103.84
θ_g	99.85^o	75.61^o	30.16^o

Table T 2. TMP unit cell dimensions in terms of the operating wavelength.

$Parameters$	$f_{low} = 1.22GHz$	$f_{high} = 30GHz$
X	$0.27\lambda_{low}$	$6.63\lambda_{high}$
Y	$0.32\lambda_{low}$	$7.88\lambda_{high}$
H	$0.18\lambda_{low}$	$4.32\lambda_{high}$

where $\vec{H}_{total} = \vec{H}^{incident} + \vec{H}^{scattered}$, $\vec{E}_{total} = \vec{E}^{incident} + \vec{E}^{scattered}$, ρ_s and $\vec{J}_s$ are the electric surface charge density and the electric current density, respectively, and μ and ε are the magnetic permeability and electric permittivity of the medium, respectively. Equation (79) shows that, any magnetic field that is tangential to a conductive surface excites equivalent electric currents that flow on this surface. If the surface is purely conductive (e.g., PEC), these currents radiate back the entire energy of the incident field and this is indeed what we observe when we have a perfect conductor. However, when the surface is resistive, like the absorber in our case, these currents are absorbed and no radiation occurs. On the other hand, as we can see from (81), any magnetic field that is perpendicular to a conductive surface is always reflected back; $\hat{n} \cdot \mu\vec{H}_{total} = 0 \rightarrow \vec{H}^{incident} = -\vec{H}^{scattered}$. This is exactly what we see with the TE impinging waves. As the angle of incidence θ increases, the magnetic field component becomes closer to be perpendicular to the aperture of the absorber, resulting into strong reflection. Figures S7c and S7d show the simulated reflectivity of the TMP absorber for different angles of incidence for the case of broadside illumination for both the TE and TM modes. To also, prove that weak or strong equivalent electric currents are excited on the aperture of our absorber due to the impinging TE and TM waves as the angle of incidence changes, we choose to plot the electric current density of both types of waves (TE and TM) at a specific frequency of operation and at two different angles of incidence where the reflectivity (or equivalently the absorptivity) changes. Namely, for a TE impinging wave, as we can see from Figs. S7e and S7g, the electric current that flows on the aperture of the absorber due to an impinging wave with angle of incidence ($\phi = 0^o$, $\theta = 30^o$), is much stronger compared to the electric current that flows on the aperture of the absorber due to an impinging wave with angle of incidence ($\phi = 0^o$, $\theta = 60^o$); notably, the reflectivity in the first case (see Fig. S7c) is lower than the reflectivity of the latter case. Equivalently, for all the TM mode responses, the reflectivity is low because, strong electric currents flow on the aperture of our absorber (see Figs. S7f and S7h).

2.4 Comparison of TMP to Hexagonal Honeycomb Arrays

The TMP and hexagonal honeycomb are analyzed for three different materials using ANSYS Workbench 2021 R2 Mechanical. The properties of these materials are shown in Table T3. Figure S8 shows the hexagonal honeycomb keeping the same x- and y-dimensions as in the TMP, where

Table T 3. Material Properties used for FEA.

Material	Modulus of Elasticity (E) (GPa)	Poisson's Ratio (ν)	Yield Strength (σ_y) (MPa)	Reference
Polyimide	3.8	0.34	89	[6,7]
Aramid Paper	7.7	0.4	47	[8]
Polylactic Acid (PLA)	3.5	0.36	21	[9,10,11]

$$x = 4m\sin\left[2\tan^{-1}(\tan\alpha\cos\left(\frac{\gamma_1}{2}\right)\right] + \frac{3}{2}d\cos\left(\frac{\gamma_1}{2}\right) \quad (83)$$

$$y = 4\left[2l + m\cos\left(2\tan^{-1}\left(\cos\left(\frac{\gamma_1}{2}\right)\tan\alpha\right)\right] \quad (84)$$

A regular hexagon does not exactly match the aspect ratio of the selected TMP. Figure S8 illustrates this mismatch of aspect ratio. The TMP array with dimensions of the rectangular area that encompasses it (B and W) is shown in Fig. S8a. Figure S8b shows the hexagonal honeycomb array with 3 full hexagons (equivalent to TMP array shown in Fig. S8b) spanning the W direction overflows outside the constant rectangular area in the B direction. Figure S8c shows the hexagonal honeycomb array with 3 full hexagons spanning the B direction (rotated 90 degrees and scalled) overflows outside the constant rectangular area in the W direction. All the associated parameters are given in Table T4 for both TMP and Hexagonal Honeycomb.

References

1. Rozanov, K. Ultimate thickness to bandwidth ratio of radar absorbers. *IEEE Transactions on Antennas Propag.* **48**, 1230–1234, DOI: 10.1109/8.884491 (2000).
2. Balanis, C. A. Advanced engineering electromagnetics. *John Wiley Sons Comp., Hoboken* (2012).
3. Yasuda, H. & Yang, J. Reentrant origami-based metamaterials with negative poisson's ratio and bistability. *Phys. Rev. Lett.* **114**, 185502, DOI: 10.1103/PhysRevLett.114.185502 (2015).
4. Yasuda, H., Yein, T., Tachi, T., Miura, K. & Taya, M. Folding behaviour of tachi-miura polyhedron bellows. *Proc. Royal Soc. A: Math. Phys. Eng. Sci.* **469**, 20130351, DOI: 10.1098/rspa.2013.0351 (2013).
5. Pozar, D. M. Microwave engineering. *John Wiley Sons Comp., Hoboken* (2012).
6. DuPont. Dupont™ kapton® summary of properties. www.dupont.com. Accessed: May, 2021.
7. Plastics, L. Technical data sheet - polyimide. www.laminatedplastics.com. Accessed:October, 2021.
8. Beaumont, P., Soutis, C. & Hodzic, A. *Structural integrity and durability of advanced composites: Innovative modelling methods and intelligent design* (Woodhead Publishing, 2015).
9. Farah, S., Anderson, D. G. & Langer, R. Physical and mechanical properties of pla, and their functions in widespread applications—a comprehensive review. *Adv. drug delivery reviews* **107**, 367–392 (2016).
10. SD3D. Sd3d pla technical data sheet. www.sd3d.com. Accessed:October, 2021.
11. Overture. Overture pla technical data sheet. www.overture3d.com. Accessed:August, 2021.

Table T 4. Parameters of the Tachi-Miura Polyhedron and resulting parameters of the hexagonal honeycomb.

**Tachi-Miura Polyhedron**	
Parameter	Value (units)
l	20 (mm)
m	40 (mm)
d	33.2 (mm)
α_b	57.2 (deg)
γ_1	80 (deg)
Area	24321 (mm^2)
**Hexagonal Honeycomb**	
Parameter	Value (units)
a	34.2077 (mm)

Table T 5. Specifications of horn antennas and network analyzers with corresponding frequency ranges.

Frequency (GHz)	Horn Antennas	Network Analyzers
1 GHz to 6 GHz	An ETS Lindgren 3115 (0.75 GHz - 18 GHz) and an SAS-571 (0.75 GHz - 18 GHz)	Agilent FieldFox VNA $N9923A$ series (2 MHz - 6 GHz)
6 GHz to 18 GHz	A pair of A-InfoMW $LB-60180-SF$ (6 GHz - 18 GHz)	Agilent $N5225A$ series PNA (10 MHz - 50 GHz)
18 GHz to 30 GHz	A pair of A-InfoMW $LB-180400-KF$ (18 GHz - 40 GHz)	Agilent $N5225A$ series PNA (10 MHz - 50 GHz)

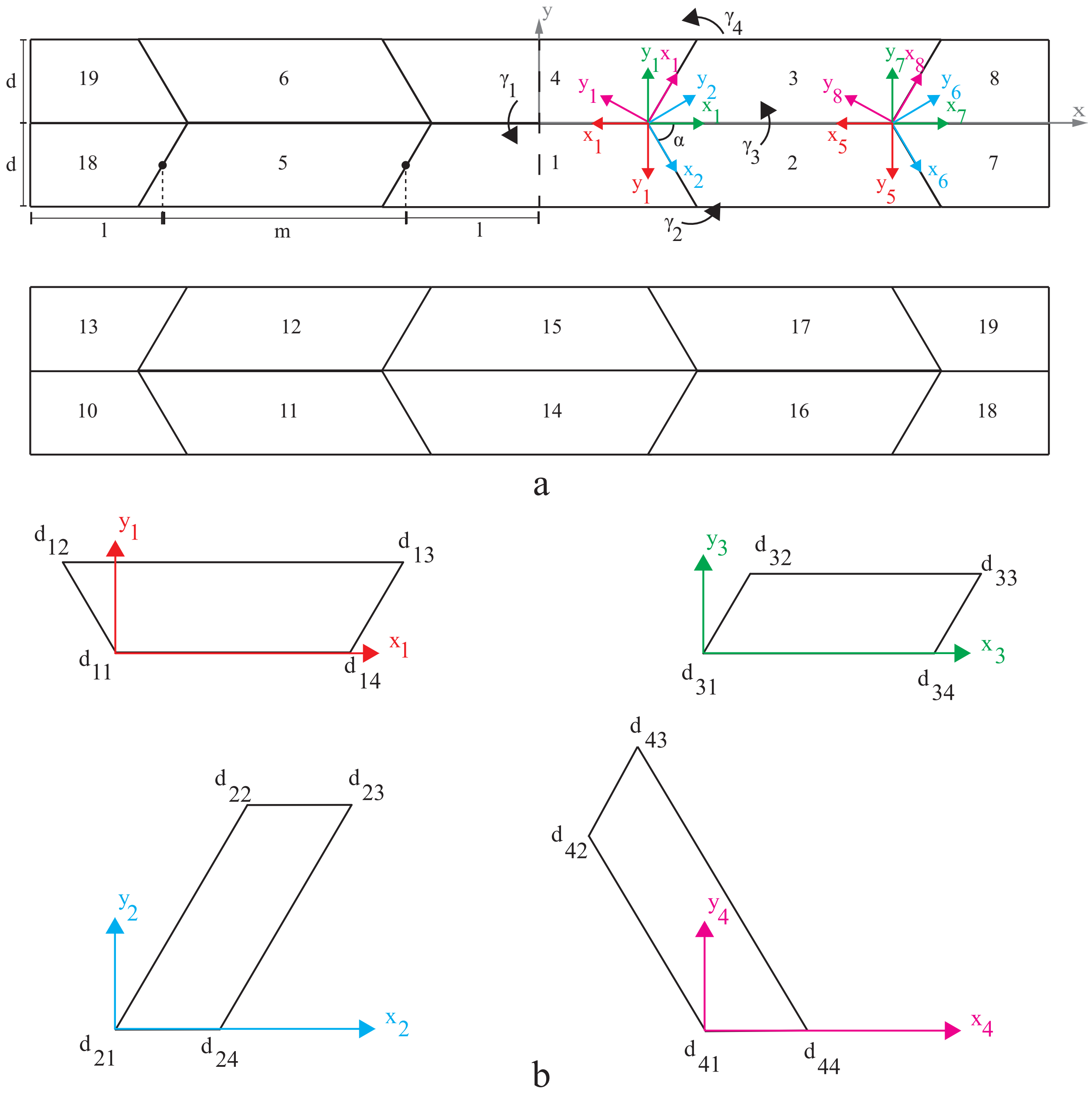

Figure S 3. (a) The front and back panels of the TMP. (b) The panels located at the first vertex from a. The local coordinate systems for each panel are shown in the same color as in a. d_{ij} is the coordinate of the jth point of ith panel.

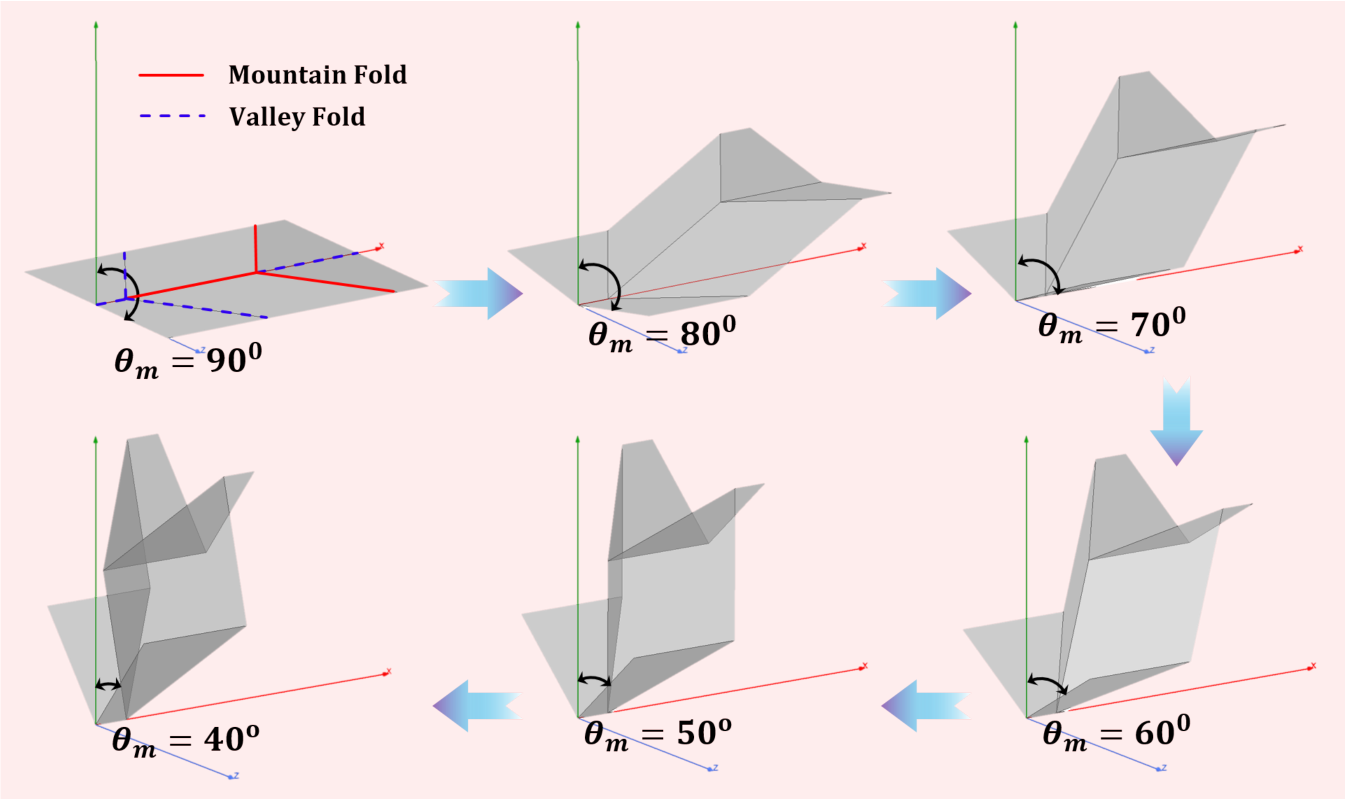

Figure S 4. Folding steps of a flat (i.e., unfolded) panel to form the building block of the TMP unit cell, which is shown in Fig. S5.

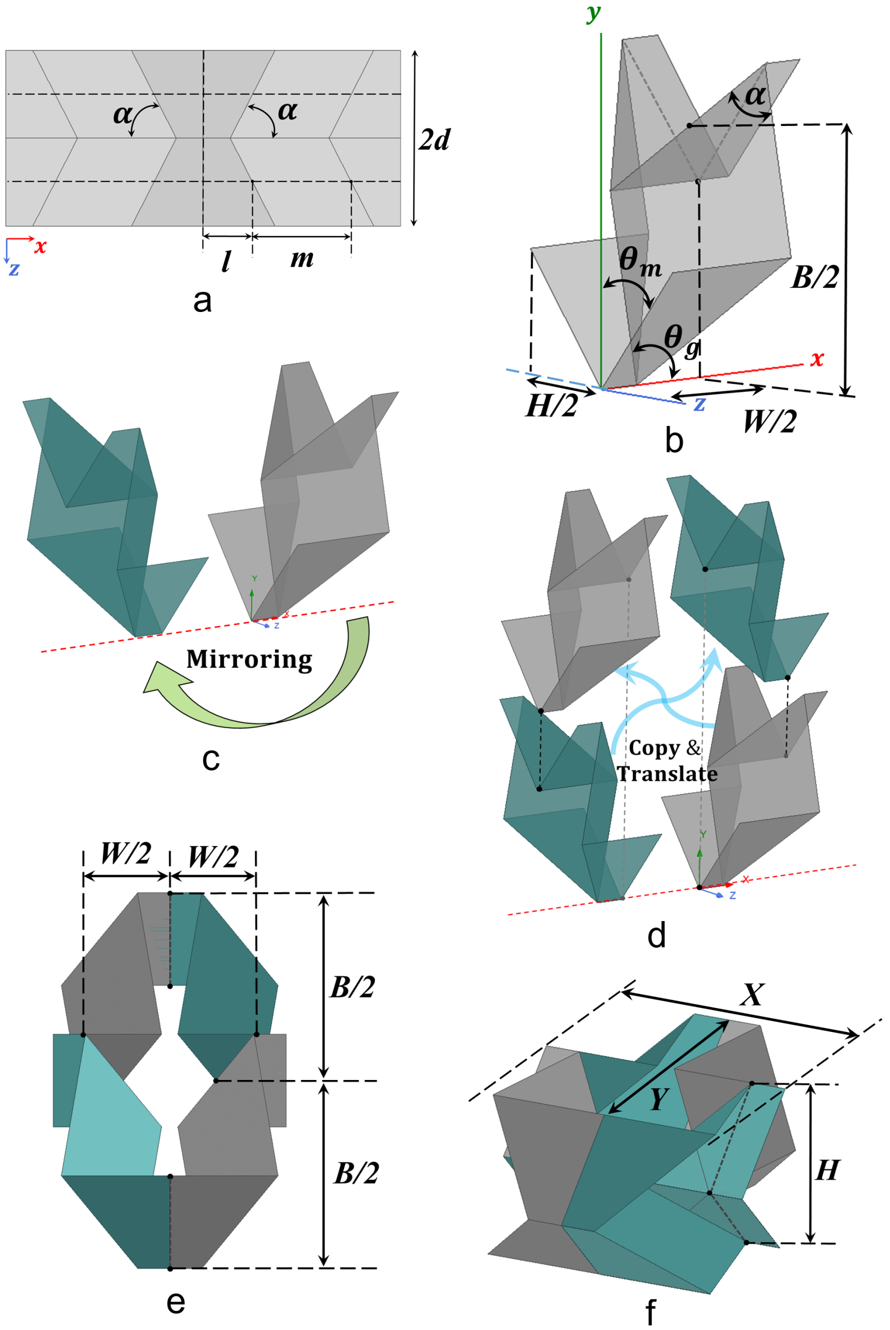

Figure S 5. TMP unitcell geometry. (a) Flat (i.e., unfolded) front panel of TMP unitcell with $\alpha = 57.2^o$, $m = 40$ *mm*, $d = 33.62$ *mm*, and $l = 20$ *mm*. (b) Folded configuration of flat front panel for $\theta_m = 40^o$, $\theta_g = 99.85^o$. (c) Mirroring of single folded panel. (d) Copy and translate operations of single folded panels to construct our TMP unit cell. (e) Top view of TMP unit cell, and (f) Perspective view of TMP unit cell.

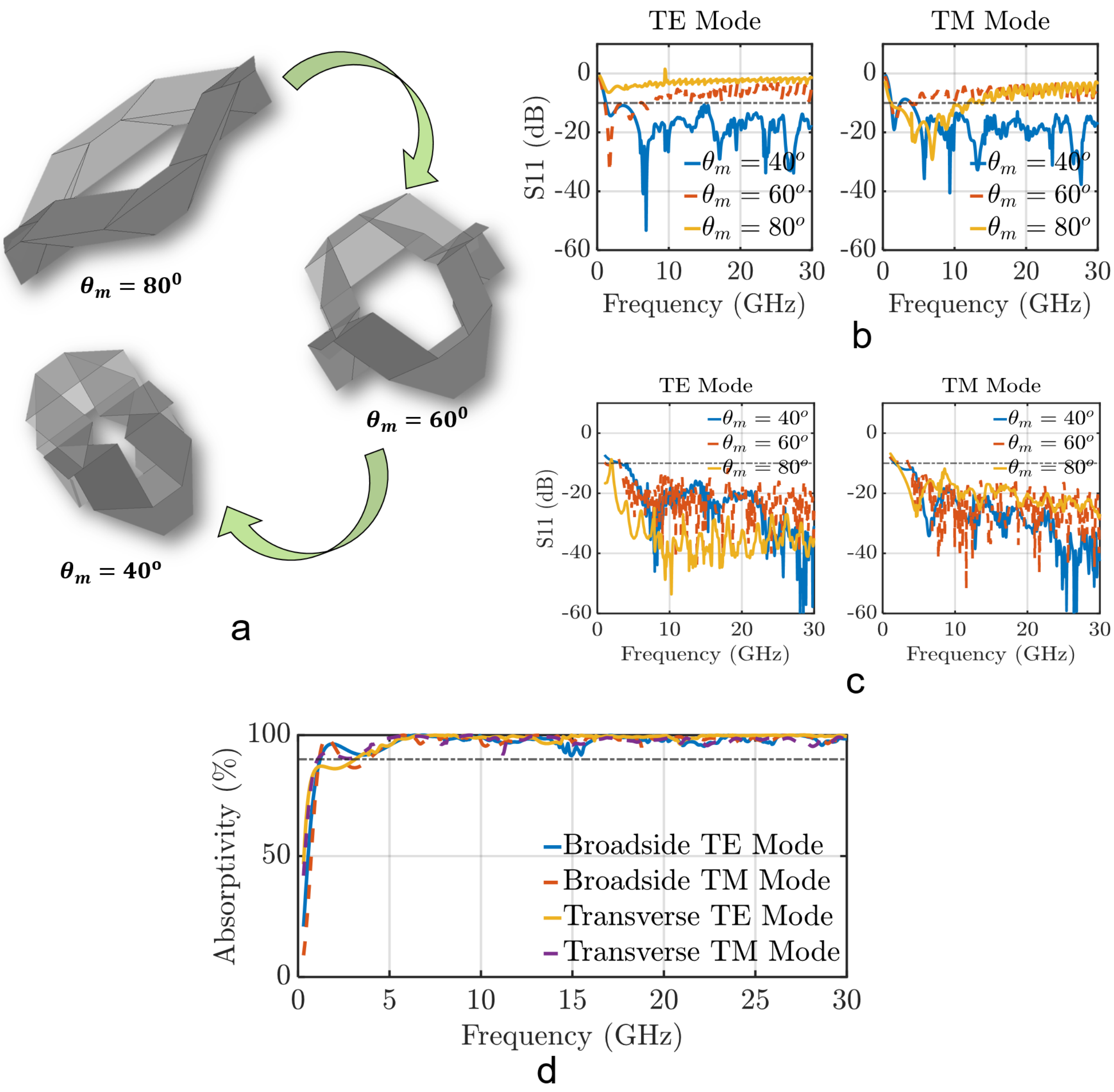

Figure S 6. TMP absorber analysis. (a) TMP unit cell at different folding angles. (b) Reflectivity response for different folding angles for broadside illumination. (c) Reflectivity response for different folding angles for transverse illumination. (d) Absorptivity for broadside and transverse illuminations for folding angle $\theta_m = 40^o$.

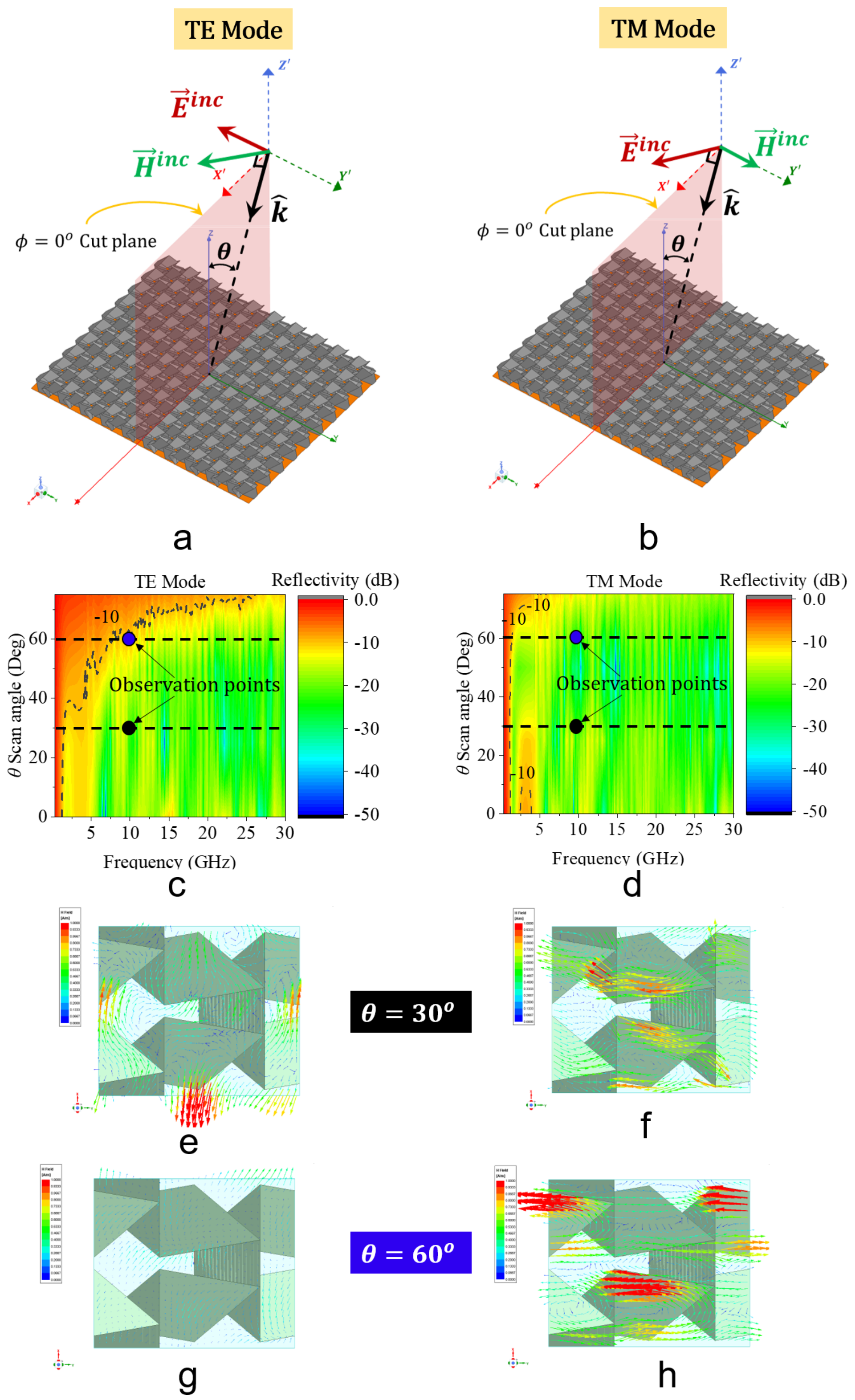

Figure S 7. Incident wave at an arbitrary angle θ for both (a) TE and (b) TM modes. Simulated reflectivity of TMP absorber for different angles of incidence for broadside illumination: (c) TE mode, (d) TM mode. Current distributions of the TMP absorber for the frequency of operation $f = 10$ GHz and for impinging waves with angles of incidence ($\phi = 0^o$, $\theta = 30^o$) and ($\phi = 0^o$, $\theta = 60^o$) for both TE and TM modes in (e), (g), and (f), (h), respectively.

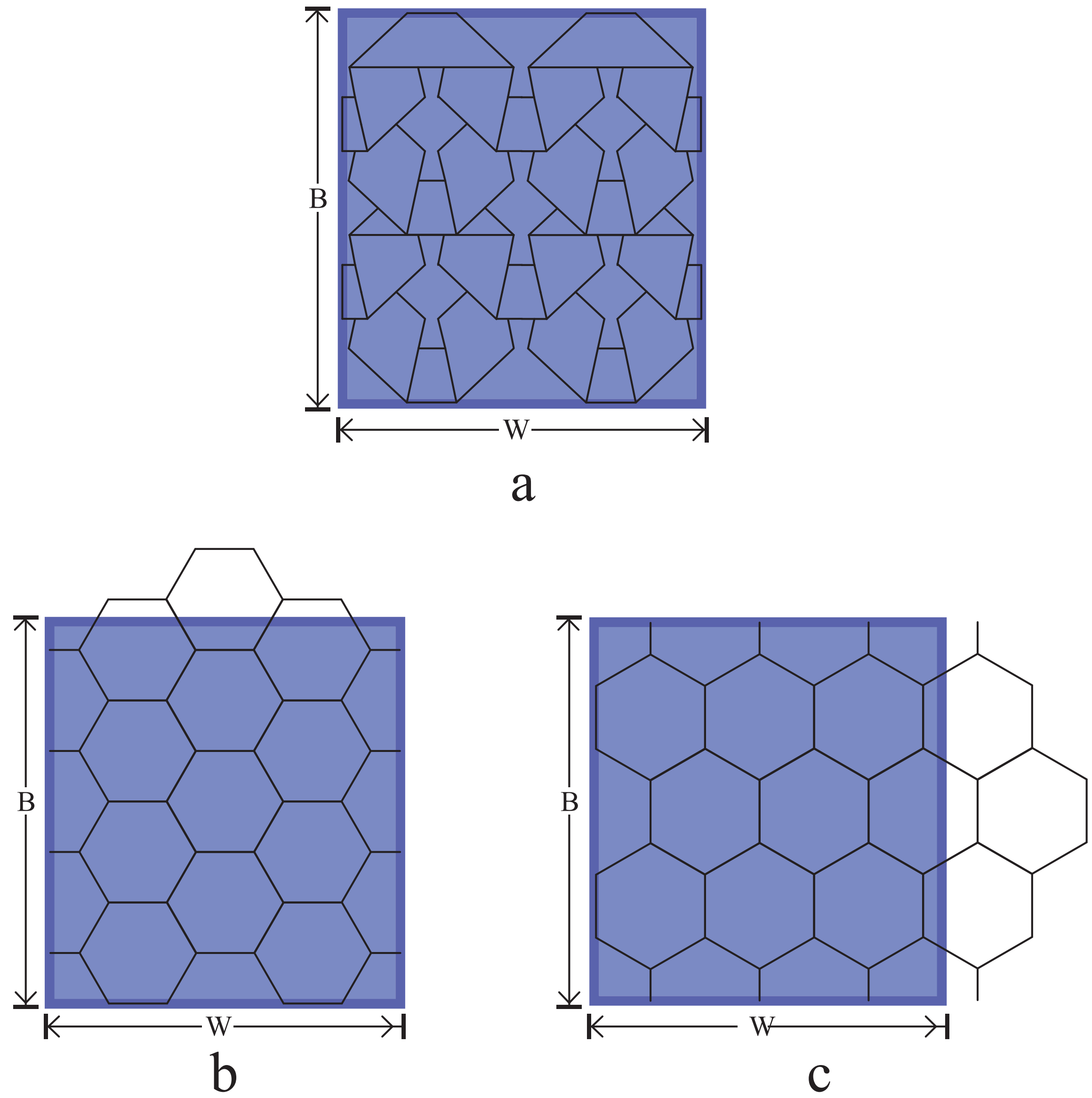

Figure S 8. (a) Unit TMP array with dimensions of the rectangular area that encompasses it (*B* and *W*). A regular hexagon does not exactly match the shape of the selected TMP. (b) The hexagonal honeycomb array with 3 full hexagons spanning the *W* direction overflows outside the constant rectangular area in the *B* direction. (c) The hexagonal honeycomb array with 3 full hexagons spanning the *B* direction overflows outside the constant rectangular area in the *W* direction.
